# The impact of hospital accreditation on the quality of healthcare: a systematic literature review

**DOI:** 10.1186/s12913-021-07097-6

**Published:** 2021-10-06

**Authors:** Mohammed Hussein, Milena Pavlova, Mostafa Ghalwash, Wim Groot

**Affiliations:** 1 Department of Health Services Research, CAPHRI, Maastricht University Medical Centre, Faculty of Health, Medicine and Life Sciences, Maastricht University, Maastricht, The Netherlands; 2 Department of Hospitals Accreditation, Saudi Central Board for Accreditation of Healthcare Institutions (CBAHI), Riyadh, Saudi Arabia; 3 Top Institute Evidence-Based Education Research (TIER), Maastricht University, Maastricht, The Netherlands

**Keywords:** Accreditation, Hospitals, Quality of health care, Health services

## Abstract

**Background:**

Accreditation is viewed as a reputable tool to evaluate and enhance the quality of health care. However, its effect on performance and outcomes remains unclear. This review aimed to identify and analyze the evidence on the impact of hospital accreditation.

**Methods:**

We systematically searched electronic databases (PubMed, CINAHL, PsycINFO, EMBASE, MEDLINE (OvidSP), CDSR, CENTRAL, ScienceDirect, SSCI, RSCI, SciELO, and KCI) and other sources using relevant subject headings. We included peer-reviewed quantitative studies published over the last two decades, irrespective of its design or language. Following the Preferred Reporting Items for Systematic Reviews and Meta-Analyses guidelines, two reviewers independently screened initially identified articles, reviewed the full-text of potentially relevant studies, extracted necessary data, and assessed the methodological quality of the included studies using a validated tool. The accreditation effects were synthesized and categorized thematically into six impact themes.

**Results:**

We screened a total of 17,830 studies, of which 76 empirical studies that examined the impact of accreditation met our inclusion criteria. These studies were methodologically heterogeneous. Apart from the effect of accreditation on healthcare workers and particularly on job stress, our results indicate a consistent positive effect of hospital accreditation on safety culture, process-related performance measures, efficiency, and the patient length of stay, whereas employee satisfaction, patient satisfaction and experience, and 30-day hospital readmission rate were found to be unrelated to accreditation. Paradoxical results regarding the impact of accreditation on mortality rate and healthcare-associated infections hampered drawing firm conclusions on these outcome measures.

**Conclusion:**

There is reasonable evidence to support the notion that compliance with accreditation standards has multiple plausible benefits in improving the performance in the hospital setting. Despite inconclusive evidence on causality, introducing hospital accreditation schemes stimulates performance improvement and patient safety. Efforts to incentivize and modernize accreditation are recommended to move towards institutionalization and sustaining the performance gains.

**PROSPERO** registration number CRD42020167863.

**Supplementary Information:**

The online version contains supplementary material available at 10.1186/s12913-021-07097-6.

## Background

“To Err is Human,” a landmark report that was published by the Institute of Medicine (IOM) in 1999 [1], recommended reinforcement of quality and safety in healthcare [2]. The report suggested that quality is multifaceted and quality assessment is one of the driving forces to establish performance improvement [3, 4]. In response, various approaches have been employed globally to regulate healthcare quality internally and externally [5]. External review systems facilitate organizational change, enhance the quality of services, and strive toward quality standards [6]. Accreditation has been cited as the oldest and most common strategic external quality assessment tool in healthcare [7, 8].

It should be acknowledged that the embryonic seeds of hospital accreditation were planted a century earlier by the American College of Surgeons [9]. Since then, hospital accreditation programs have thrived ubiquitously and become an integral part of healthcare quality systems [10–12]. In the last two decades, many countries have adopted or adapted hospital accreditation systems [13].

Accreditation refers to the external peer review that evaluates a healthcare organization’s compliance against pre-defined performance standards [14], with the ultimate aim to improve healthcare quality [15]. It is overseen by various governmental and non-governmental entities, using different modalities in voluntary or mandatory approaches. The scope of accreditation can cover the entire healthcare facility or only a specialty or even a sub-specialty [6, 16]. Several leading international healthcare organizations have viewed accreditation as a valid marker of quality [12] and discussed the effectiveness of using accreditation standards as a tool to enhance organizational and clinical performance [17–19]. Nevertheless, the available evidence in the literature supporting this assumption remains scarce.

Despite the ostensible promising effect of healthcare accreditation [20, 21], the literature presents a complex view of its impact [22]. The legitimacy concerns about accreditation are due to the scant of high-quality trials and conflicting reported results [23–25]. Contradictory findings have generated inconsistency in the conclusions of previously published reviews [6, 12, 13, 23, 26–35]. On the one hand, positive impacts of hospital accreditation on organizational culture [12, 32, 34], clinical practice, organizational performance [23], clinical leadership, patient safety systems [28], quality of services [29], care delivery process [30], and efficiency [35] have been demonstrated. On the other hand, several reviews reported insufficient evidence pertaining to the impact of accreditation on measurable changes in quality of care [12], health outcomes [26], patient satisfaction [31], and economic outcomes [13, 26, 34]. For instance, Greenfield and Braithwaite [13] present diverging findings on the impact of accreditation as the effect was limited to promoting change and professional development, while on other impact categories such as quality measures, financial impact, and public disclosure results were inconclusive. In addition, some reviews questioned the cost-effectiveness of accreditation [6, 32, 33].

Previously published accreditation reviews included the impact of specialty [30] or disease-specific [34] accreditation programs which could dilute the overall impact of hospital accreditation, used stringent inclusion designs that could limit its contribution room [6, 12], restricted search languages, or overlooked several important relevant studies [35]. This review has overcome such hindrances and aimed to identify and analyze the evidence on the impact of hospital accreditation.

## Methodology

Our review was based on the Preferred Reporting Items for Systematic Reviews and Meta-Analyses (PRISMA) guidelines [36], presented in Additional file 1. We verified that there was no running or completed systematic review like ours in Prospero and Health Systems Evidence (HSE) database at the commencing phase. Thereafter, we registered the protocol of our review as PROSPERO ID: 167863 on 04-Feb-2020 to avert “HARKing” [37].

### Databases and search terms

Electronic bibliographic databases were searched systematically to retrieve relevant publications using relevant subject headings and controlled vocabulary terms, as shown in Additional file 2. Databases include; PubMed, CINAHL, PsycINFO, EMBASE, MEDLINE (OvidSP), ScienceDirect, Cochrane Database of Systematic Reviews (CDSR), Cochrane Central Register of Controlled Trials (CENTRAL), and Web of Science, including Social Sciences Citation Index (SSCI), Russian Science Citation Index (RSCI), SciELO Citation Index, and KCI-Korean Journal Database. The search reported here was effectuated by the primary author on 18-Feb-2020 after being reviewed by a specialist librarian.

Additionally, we searched Google Scholar using keywords in different combinations, including accreditation, hospital, quality, impact, and healthcare services. Furthermore, we scanned the websites of the most popular accreditation entities for additional papers that we might have overlooked.

### Screening and eligibility assessment

We included full-text publications that evaluated the impact of overall hospital accreditation programs on the quality of healthcare services in the last two decades (i.e., since “To Err Is Human”) from January 2000 – February 2020. All quantitative studies were included irrespective of their design. No language restriction was added. Following the search, titles and abstracts were retrieved and uploaded into the bibliographic reference management software EndNote X9, and deduplicated. Thereafter, two authors (MH, MG) independently screened all titles and abstracts to identify potentially relevant articles and read the full text of relevant studies to assess eligibility. Studies were assessed for eligibility using the PICO criteria [38]: population— all types of hospitals; intervention— all types of overall accreditation; comparison— unaccredited hospitals, before-and-after, or different accreditation levels; outcomes— measurable impacts on the structure, process, or outcome parameters. At any stage, disagreement between the two authors was reunited by consensus or arbitration by a third author (MP).

We excluded unpublished/unindexed studies, review articles, or studies published in an “abstract” format. Studies conducted in healthcare settings other than hospitals, studies that evaluated the impact of accreditation on a specialty or disease-specific, or examined accreditation preparation cost were excluded. In addition, studies that assessed the perceived benefits of accreditation have been excluded. However, to evaluate the impact from different perspectives, comparative studies that examined accreditation effects on self-reported subjective outcome parameters (e.g., patient satisfaction, job stress) using a validated instrument were included.

A kappa inter-rater reliability (IRR) test was conducted to assess full-text assessment reliability [39, 40]. We randomly selected and matched a sample of 50 studies that were evaluated for inclusion by the two reviewers. Four differences were identified, which resulted in kappa 0.81, indicating a high agreement level.

### Data extraction

Studies that met our inclusion criteria were interrogated independently by two authors using a standardized data extraction form, and its references were screened (i.e., snowballing) for additional potentially relevant studies. Details on the research designs, goals, findings, and conclusions were extracted and compiled for analysis. Occasionally, when information insufficiency hindered data extraction, it was solicited from the corresponding author. All relevant non-English-language studies were translated through Google Translate, which has been cited as a reliable tool for translating papers published in languages other than English in systematic reviews [41, 42]. For authenticity, we e-mailed the data extracted from the included non-English studies to the corresponding author for verification and stipulated obtaining confirmation for inclusion. Studies that did not meet our inclusion requirements were summarized along with the reason for exclusion, and records were preserved for audit trail purposes.

### Quality assessment

In this review, the methodological precision of included publications was assessed using Hawker et al [43]. framework as it provides an appropriate unified scale for heterogeneous study designs. The instrument consists of nine items (namely; abstract and title, introduction and aims, method and data, sampling, data analysis, ethics and bias, findings, transferability, and implications and usefulness), each scored on a 4-point scale (1 = good; 2 = fair; 3 = poor; 4 = very poor). The overall grade was judged based on the average score of these items (1.00–1.49 = good, 1.50–2.49 = fair; 2.50–3.49 = poor; 3.50–4.00 = very poor) [44].

For each included study, the coders (MH, MG) independently assessed the methodological quality, assigned an appropriate score, and calculated the overall grade accordingly. To test the assessment credibility, a kappa IRR test was employed using 20 randomly selected assessed studies. A crosswalk between decisions revealed two disparities, resulting in kappa 0.8, which indicates a trustworthy agreement level [39, 40].

### Analysis

For text mining [45], extracted data were synthesized and presented narratively using the thematic analysis [46]. The effects were categorized into six impact themes, that were reported in part or entirely in previous reviews [6, 12, 13, 26, 29, 32] and models [47]. In this review, the impact of accreditation was defined as a measurable marked effect that the accreditation process demonstrated, positively or negatively. The impact was judged to be positive if all or most of the results were significantly advantageous, negative if all or most of the results were unfavorable, or neutral when no real change due to accreditation was identified [26]. The impact themes were: changes in organizational culture and management; changes at professionals’ level; changes at the patient level; changes in patient clinical outcomes; changes in performance measures; and changes in economic outcomes. Each study was classified under one or multiple outcome themes.

## Results

### Search results

**Fig. 1:**
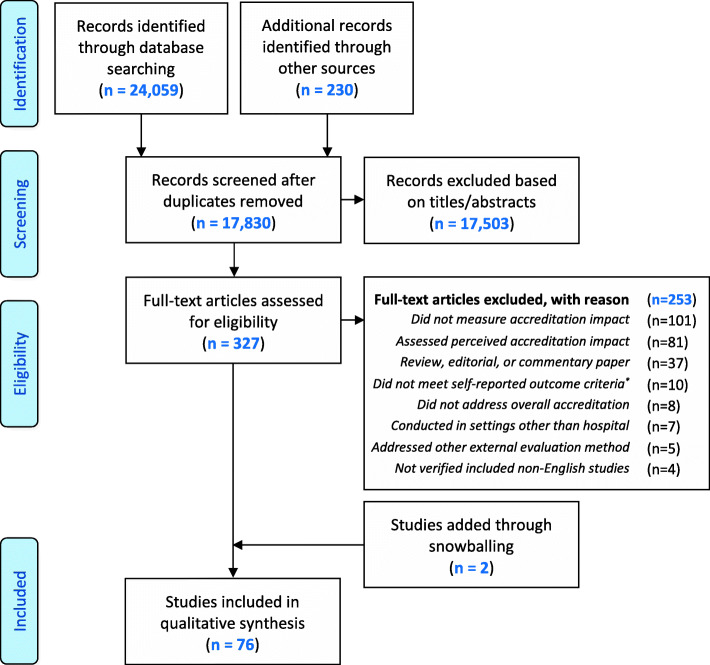
PRISMA flow diagram illustrating review process. * Self-reported subjective outcome parameter through a validated instrument, using comparative design

Our search identified 17,830 publications. Based on the title and abstract screening, 327 articles were deemed potentially eligible and retrieved for full-text review. Of these, 74 studies matched our inclusion criteria. This included seven non-English studies verified by their authors, while four other non-English studies were excluded due to no response to our verification request. Two additional studies were identified through screening the references of included articles, which yielded 76 studies for critical appraisal and analysis (see Fig. 1).

### Features of the included studies

Additional file 3 summarizes the key findings of all studies included in our review. During the last decade, there has been a notable flourish in the number and spectrum of studies evaluating the impact of accreditation in the literature. Almost three fourths (*n* = 52) of the included studies were published during the last five years (2015–2019). The majority of studies were in English (*n* = 69). The seven verified and analyzed non-English studies were published in Persian, Danish, Korean, and Hungarian.

Included studies were conducted in 22 countries representing all inhabited continents. The highest number of studies were from the USA (*n* = 11) and Brazil (*n* = 9). Two studies were multinational, conducted in European hospitals [19, 28]. Studies evaluated the impact of 23 accreditation programs. The most studied scheme was the Joint Commission International Accreditation (JCIA) (*n* = 14). Twenty-one studies (28%) assessed the impact of accreditation in a single hospital, while the range was up to 4400 hospitals.

### Assessment of the methods used

In our review, many studies have a cross-sectional design (*n* = 29). A before-after design was utilized in 30 studies. Cohort and quasi-experimental designs were employed in 12 and 14 studies, respectively. Notably, only one randomized clinical trial (RCT) was found and included [48]. This level of evidence may indicate an association between accreditation and performance measures; however, causal inferences should be made with considerable caution. A meta-analysis was not possible with these observational designs and the modest methodological consistency.

The appraisal of the included studies showed that 32, 37, and 7 studies were of good, fair, and poor methodological quality, respectively. Studies with poor methodological quality have shown a positive [49–51] (*n* = 3) or neutral [52–55] (*n* = 4) accreditation effect; albeit, their findings should be scrutinized with care. Our narrative analysis disregarded these studies to avoid jeopardizing the conclusion. This seemed unlikely to alter the review findings.

### The impact themes

Included papers were thematically clustered into six impact themes (see Table 1). Two themes, namely “changes in patient clinical outcomes” and “changes in performance measures,” captured more than 60% of included publications. Although our themes are collectively exhaustive, they are not mutually exclusive as 16% (*n* = 12) of the studies examined the impact of accreditation on at least two measures in separate themes.

**Table 1:** Methodological quality ratings and impact directions of included studies (*n* = 76)

Themes	Definition and Examples	Related Studies Cited as per the Reference List	Methodological Quality			Impact Direction of Good & Fair Studies		
			Good	Fair	Poor	Positive	Negative	Neutral
**Changes in organizational culture and management (*n*** **= 5)**	Demonstrated as a significant quantitative hospital managerial or cultural change (e.g., safety culture, communication)	56–60	1	4	0	4	0	1
**Changes at the professionals’ level (*n*** **= 10)**	Demonstrated as changes in professionals’ self-reported outcome parameters (e.g., job stress, job satisfaction)	49, 59, 61–68	3	6	1	1	4	4
**Changes at the patient level (*n*** **= 14)**	Demonstrated as a measurable change in self-reported subjective outcome parameters from a patient and user perspective (e.g., patient satisfaction, patient experience)	21, 48, 53, 55, 69–78	6	6	2	3	2	7
**Changes in patient clinical outcomes (*n*** **= 24)**	Demonstrated as a statistically significant change in patient health outcome measures (e.g., mortality rate, length of stay)	8, 21, 25, 50–53, 79–95	8	12	4	15	0	5
**Changes in the performance measures (*n*** **= 28)**	Demonstrated as a statistically significant change in clinical performance measures (e.g., hand hygiene compliance, medication utilization)	8, 19, 28, 48, 51, 54, 60, 68, 79, 87, 90, 96–111	14	12	2	18	0	8
**Changes in economic outcomes (*n*** **= 8)**	Demonstrated as quantifiable changes in financial or economic outcome parameters (e.g., efficiency, profitability)	83, 90, 112–117	4	4	0	5	1	2

#### Changes in organizational culture and management

The impact of hospital accreditation on organizational culture and management was examined quantitively in five studies [56–60]. Several studies have examined the effect of hospital accreditation on safety culture through self-reported surveys. Most [56–58] but not all found a strong link between both [59]. Accreditation positively affects perceived patient safety culture [56], safety climate toward medication error reporting [57], and organizational culture as manifested by a less hierarchical culture and more group and developmental culture [58]. On the contrary, a recent study did not detect changes in the safety management culture from the nurses’ perspective after accreditation [59].

#### Changes at the professionals’ level

Our review identified ten studies that assessed the impact of accreditation on self-reported parameters such as job stress, job satisfaction, and work environment [49, 59, 61–68], five being before-after studies, while a comparative approach between accredited and non-accredited hospitals was used in the remaining. Authors found negative (*n* = 4) or no impact of accreditation (*n* = 4) at the professionals’ level, particularly for nurses who were the selected subjects in 7 studies.

Studies reported a consistently negative impact of hospital accreditation on professionals’ perceived job stress. For example, in 4 studies, accreditation was associated with higher job stress as perceived by health professionals [59, 61–63]. In addition to stress, Elkins et al. [63] reported higher anxiety and depression among nurses during the accreditation preparation phase, as well as a significant improvement in job satisfaction and sleep function post-accreditation. However, due to the limited research available, it remains uncertain if accreditation affects job satisfaction or the working environment.

#### Changes at the patient level

Only 14 studies that assessed the impact of hospital accreditation on measurable patient-reported outcome parameters were found [21, 48, 53, 55, 69–78]. Studies mainly used an observational cross-sectional design (*n* = 12).

Despite the widely held belief that accreditation contributes to improving patient satisfaction and experience, most findings provide little evidence to support whether accreditation status or ratings are measurably linked to patient satisfaction and experience in a meaningful way. Multiple studies that compared accredited with non-accredited hospitals [21, 48, 70, 71, 77, 78] or accredited hospitals at different accreditation levels [69, 72] did not find any association. For instance, Sack et al [77, 78] did not find a link between accreditation and patients’ perception of better quality, reflected by their recommendation rates of the institutions at the hospital level or the cardiology unit level.

#### Changes in patient clinical outcomes

Interestingly, around one third (*n* = 24) of the included studies examined the impact of hospital accreditation on patient outcomes [8, 21, 25, 50–53, 79–95]. Of these, 75% have been published since 2015 as an obvious response to previous appeals to investigate accreditation effects on clinical outcomes. Overall, the results showed a clear trend toward a positive relationship between accreditation and clinical outcome. Studies reported having (*n* = 15) or lacking (*n* = 5) positive effects on clinical outcomes, whereas none of the studies concluded having an overall negative impact. In-hospital mortality rate (*n* = 13) and the patient’s length of stay (*n* = 12) were studied most.

Comparative studies showed a positive effect of accreditation on mortality rates at various accreditation stages [79–84]. However, these studies were restricted to two accreditation schemes, namely, The Joint Commission on Accreditation of Healthcare Organizations (JCAHO) in the USA and Danish healthcare quality program (DDKM in Danish: den danske kvalitetsmodel) in Denmark, which may hinder generalization. For example, relative to hospitals with low [82, 83] or persistently low [84] accreditation standards compliance, patients treated in high compliance hospitals were found to have significantly lower mortality. Dissimilarly, such a relationship was not identified in other studies [8, 21, 85–88].

Several studies consistently indicated a lack of relationship between accreditation and hospital 30-day readmission rate in various contexts [21, 84, 89, 90], whereas other studies presented contradictory effects on healthcare-associated infections [25, 85, 91, 92]. However, studies reported a consistently positive impact of accreditation on hospital [84, 86, 89, 93] and departmental [91, 94, 95] patient length of stay.

#### Changes in the performance measures

There is plausible evidence that hospital accreditation promotes service quality. Consequently, improvement in structure and process performance measures could be expected [21, 83]. The impact of accreditation on performance measures was the largest topic (*n* = 28) explored in our review [8, 18, 19, 28, 48, 51, 54, 60, 68, 79, 87, 90, 96–111]. Despite the complexity and cyclicality of accreditation effects on performance measures, about three-fourths (*n* = 18) of the analyzed studies showed a positive effect of accreditation on service quality at organizational and departmental levels.

Although the only included RCT reported no or low association between accreditation and quality indicators [48], the methodological quality of this study was fair but not good enough to generalize this finding. It is noteworthy that several quasi-experimental and prospective longitudinal studies reported significant positive effects of accreditation on various quality of service aspects [8, 60, 96–99]. Accumulated evidence showed that longitudinal participation in accreditation translated into higher standards compliance [60], adherence to recommended guidelines [97], enhancement in structural and process elements [19, 28], and sustained change [98]. For instance, in a stepped-wedge multi-level study, accreditation resulted in significant improvement of various processes that did not meet the target performance during the 6-month period prior to the accreditation survey [99]. Participation in accreditation has shown tangible benefits in performance measures linked to acute myocardial infarction [79, 100], heart failure, and pneumonia [100]. Nevertheless, some studies have found that accreditation is not associated with hand hygiene compliance [101], medication administration error rates [102], and other performance measures [87, 103, 104].

#### Changes in economic outcomes

A total of eight studies evaluating the economic effects of accreditation have been included [83, 90, 112–117]. Most of them (*n* = 5) showed a positive impact on various economic outcomes, particularly healthcare efficiency.

Apart from estimating the cost of accreditation, which varied dramatically between countries and programs, accreditation was shown to have a significantly favorable effect on cost reduction [90], increase in the share of outpatient revenue [83], higher productivity [112], and improved efficiency [113–115]. For example, a large retrospective longitudinal study, tracking 748 hospitals over 10 years, reported a significant positive net impact of hospital accreditation on improving the mean efficiency as estimated through bootstrapped data envelopment analysis (DEA) at accreditation year and the 2 years following [113]. Another observational study found that hospital accreditation, ceteris paribus, was associated with 119% improvement on a quality index relative to baseline data, which translated into a combined saving of US$ 593.000 in two hospitals over 3 years [90]. On the contrary, participating in accreditation programs was found to have an inverse effect on hospital efficiency secondary to higher staffing demand and investment in equipment [116]. Other studies did not detect a major impact of accreditation on operating room efficiency [117], cash-flow margin, and total cost per case [83].

## Discussion

This review has comprehensively analyzed the hospital accreditation literature during the last two decades to understand its effect on the quality of health services. In total, 76 studies have been included and assigned to a relevant impact category.

Despite the mixed views expressed, a positive accreditation effect was found in more than 55% of the included studies. Our results indicate a consistent positive accreditation effect on process-related performance measures, safety culture, hospital efficiency, and patient length of stay. In contrast, staff job stress was found to be consistently negatively affected. Heterogenous results on mortality and healthcare-associated infection hampered the drawing of firm conclusions on those outcome measures. Staff job satisfaction, patient satisfaction and experience, and 30-day readmission rate were found to be unrelated to accreditation. However, the variation in accreditation schemes [19], the inability to isolate extrinsic confounders, and diversity in hospital characteristics may influence these conclusions.

Although culture is an oft-cited reason for failure, consistent with previous studies [13, 22, 32], our review found a positive effect of accreditation on safety culture at the organizational level. However, at the individual level, accreditation has an adverse impact on professionals’ stress level [59, 61–63]. This may indicate a need for a balance between accreditation risks and benefits to encourage health practitioners’ acceptance and participation in the accreditation journey [30, 118]. Such negative consequence seems inevitable. However, awareness campaigns, leadership support, and better design of accreditation standards and processes are vital remedies that need to be considered [119].

As an extension of previous reviews [13, 31, 32, 34], our analysis did not find a correlation between accreditation and higher patient satisfaction or experience. The earlier presumption that patient satisfaction is a reverberation of hospital quality of service [120] was not confirmed in our review. While our findings support the view that accreditation is a tool that stimulates improving internal processes delivery [121], the appropriate improvement threshold for being tangible is equivocal. Likely, the answer depends on the design of the accreditation standards and processes [4, 122].

Our review found that hospital accreditation benefits appear before [56, 96], during [80], and after accreditation [97, 107]. Nevertheless, the question of the cyclicality of the impact of accreditation and how long the effect lasts is a matter of concern [16, 81, 99, 123]. For the economic outcomes, studies attribute the favorable impact of accreditation to performance improvement [90]. However, the low number of studies hindered definite conclusions. Isolating the accreditation’s financial impact from other contextual factors is challenging and may explain the paucity of studies in this domain [13, 124].

More studies on the impact of hospital accreditation are needed to elucidate part of the *jigsaw puzzle*. An argument might be that the heterogeneity in the accreditation literature and its observational nature limits its value in providing convincing conclusions on accreditation effectiveness [125]. However, the absence of firm evidence of the effects is not evidence of a lack of effect. Having realized the ethical and practical challenges of conducting randomized trials on this multifaceted process [11], observational studies appear to be of doubtless merit despite their drawbacks.

The bulk of the studies in our review used cross-sectional or two-point comparative (i.e., before-and-after) designs. Therefore, an argument could be that the observed improvement in observational studies is not necessarily attributed to the accreditation per se. However, this assumption does not rationalize abandoning what has been found already, and if observed improvements were secondary to other accreditation-driven factors, it is indeed still a win-win situation.

Our review has several strengths and limitations. This study is one of the largest systematic reviews conducted to understand the impact of hospital accreditation. The study extensively discussed the measures and aspects being addressed and affected by introducing hospital accreditation to elucidate the complex view for researchers, policymakers, and stakeholders in the accreditation field. The use of pre-decided inclusion criteria, citation indices, and broad range of databases were enablers to enhance the likelihood of identifying all relevant publications. We recognize that overlooking some studies that are not published in peer-reviewed journals is still possible. However, our comprehensive search suggests that results’ bias is unlikely. We should acknowledge that not searching the grey literature is a limitation in our review. The grey literature can provide a valuable contribution to the review and may reduce publication bias [126]. However, to maintain the validity of the results, we limited our search to studies rigorously peer-reviewed or indexed in academic journals [127]. Despite the fact that our review included evidence on accreditation effectiveness in both developing and developed countries, no distinction between these settings was made.

## Conclusion

Accreditation must be viewed as one element that complements other performance improvement strategies to achieve a tactile effect in the health system. The view must be compatible with the fact that accreditation is a “knowledge translation” intervention that aids in the integration of standards into everyday activities [128]. The advantages of accreditation outweigh potential drawbacks. However, we echo previous reviews [6, 12, 23, 32, 33, 129] in calling for further rigorous studies to investigate the impact of accreditation, particularly on economic outcomes to evaluate if the benefits genuinely justify the costs. Utilizing longitudinal designs and controlling for exogenous confounders could help detect causal conclusions of accreditation effects and enrich consequential decisions in this realm.

Our review underpins the notion that compliance with accreditation standards has multiple plausible benefits in improving the performance in hospital settings and outcomes. Despite inconclusive evidence on causality and minor unintended negative consequences of hospital accreditation, such as those on job stress, we conclude that introducing hospital accreditation stimulates performance improvement and patient safety. In synchronization with other health policies, efforts to incentivize and modernize accreditation are recommended to move towards institutionalization and sustaining the performance gains.

## Supplementary Information

**Additional file 1:.** PRISMA Checklist.

**Additional file 2:** Database Search Strategies.

**Additional file 3:** Summary of the key findings of all studies included in the review (*n* = 76).

## Data Availability

Data relevant to the study are included in the article or uploaded as Additional files. Detailed ratings of methodological quality are available upon reasonable request.

## Abbreviations

IOM: Institute of medicine
PRISMA: Preferred reporting items for systematic reviews and meta-analyses
HSE: Health systems evidence
CDSR: Cochrane database of systematic reviews
CENTRAL: Cochrane central register of controlled trials
SSCI: Social sciences citation index
RSCI: Russian science citation index
PICO: Population, intervention, comparison, outcomes
IRR: Inter-rater reliability (IRR)
JCIA: Joint commission international accreditation
RCT: Randomized clinical trial
JCAHO: The Joint commission on accreditation of healthcare organizations
DDKM: Danish healthcare quality program (in Danish: den danske kvalitetsmodel)
DEA: Data envelopment analysis
