# Supplementary material for: The impact of hospital accreditation on the quality of healthcare: a systematic literature review
Source: BMC Health Serv Res. 2021 Oct 6;21:1057. doi: 10.1186/s12913-021-07097-6 (PMC8493726; doi:10.1186/s12913-021-07097-6)
Supplement: Supplementary file 2 — Additional file 2: Database Search Strategies. [file 12913_2021_7097_MOESM2_ESM.pdf]

Additional File 2: Database Search Strategies

1) PubMed

- Searched via PubMed - NCBI (https://www.ncbi.nlm.nih.gov/pubmed/).
- Searched on 18 February 2020.
- Records retrieved: 10921

((("accreditation" [MeSH Terms] OR "accreditation" [All Fields] OR "accrediting" [All Fields] OR "accredits" [All Fields] OR "accredit" [All Fields] OR "accredited" [All Fields] OR "accreditations" [All Fields] OR "Joint Commission on Accreditation of Healthcare Organizations" [MeSH Terms] OR "joint commission on accreditation of healthcare organizations" [All Fields] OR "Joint Commission on Accreditation of Hospitals" [All Fields] OR "JCAHO" [All Fields]) **AND** ("hospitals" [MeSH Terms] OR "hospitals" [All Fields] OR "hospital" [All Fields] OR "center" [All Fields] OR "centre" [All Fields] OR "centers" [All Fields] OR "centres" [All Fields] OR "facility" [All Fields] OR "facilities" [All Fields] OR "health organizations" [All Fields] OR "healthcare organizations" [All Fields])) **AND** ("quality of health care" [MeSH Terms] OR "quality of health care" [All Fields] OR "quality" [All Fields] OR "quality improvement" [MeSH Terms] OR "quality improvement" [All Fields] OR "health services" [MeSH Terms] OR "health services" [All Fields] OR "health service" [All Fields] OR "healthcare service" [All Fields] OR "healthcare services" [All Fields] OR "health care service" [All Fields] OR "health care services" [All Fields] OR "quality assurance, health care" [MeSH Terms] OR "quality assurance, health care" [All Fields] OR "performance indicator" [All Fields] OR "performance measure" [All Fields] OR "benchmarking" [MeSH Terms] OR " benchmarking" [All Fields] OR "patient safety" [MeSH Terms] OR "patient safety" [All Fields] OR "delivery of health care" [MeSH Terms] OR "delivery of health care" [All Fields] OR "delivery of healthcare" [All Fields] OR "affect" [MeSH Terms] OR "affect" [All Fields] OR "effect" [All Fields] OR "Impact" [All Fields] OR "value" [All Fields] OR "influence" [All Fields] OR "change" [All Fields] OR "achieve" [All Fields] OR "induce" [All Fields] OR "improve" [All Fields] OR "progress" [All Fields] OR "increase" [All Fields] OR "help" [All Fields] OR "positive" [All Fields] OR "develop" [All Fields] OR "better" [All Fields] OR "advance" [All Fields] OR "raise" [All Fields] OR "rise" [All Fields] OR "enhance" [All Fields] OR "reinforce" [All Fields] OR "decrease" [All Fields] OR "negative" [All Fields] OR "reduce" [All Fields] OR "reduction" [All Fields] OR "evaluate" [All Fields] OR "evaluation" [All Fields] OR "assess" [All Fields] OR "association" [MeSH Terms] OR "association" [All Fields] OR "relationship" [All Fields] OR "perception" [MeSH Terms] OR "perception" [All Fields] OR “outcome” [All Fields] OR “result” [All Fields] OR "satisfaction" [All Fields] OR “mortality” [MeSH Terms] OR “mortality” [All Fields] OR “morbidity” [MeSH Terms] OR “morbidity” [All Fields] OR "infections" [MeSH Terms] OR "infections" [All Fields] OR "infection" [All Fields] OR "cost" [All Fields] OR "amount" [All Fields])) AND "loattrfull text"[sb] **AND** ("2000/01/01"[PDAT] : "2020/02/18"[PDAT])

SEARCH TERMS PER THEMATIC SEARCH BLOCK	
**BLOCK 1** Accreditation Block:	"accreditation" [MeSH Terms] OR "accreditation" [All Fields] OR "accrediting" [All Fields] OR "accredits" [All Fields] OR "accredit" [All Fields] OR "accredited" [All Fields] OR "accreditations" [All Fields] OR "Joint Commission on Accreditation of Healthcare Organizations" [MeSH Terms] OR "joint commission on accreditation of healthcare organizations" [All Fields] OR "Joint Commission on Accreditation of Hospitals" [All Fields] OR "JCAHO" [All Fields]
	**AND**
**BLOCK 2** Hospital Block:	"hospitals" [MeSH Terms] OR "hospitals" [All Fields] OR "hospital" [All Fields] OR "center" [All Fields] OR "centre" [All Fields] OR "centers" [All Fields] OR "centres" [All Fields] OR "facility" [All Fields] OR "facilities" [All Fields] OR "health organizations" [All Fields] OR "healthcare organizations" [All Fields]
	**AND**
**BLOCK 3** Quality of Healthcare Block:	"quality of health care" [MeSH Terms] OR "quality of health care" [All Fields] OR "quality" [All Fields] OR "quality improvement" [MeSH Terms] OR "quality improvement" [All Fields] OR "health services" [MeSH Terms] OR "health services" [All Fields] OR "health service" [All Fields] OR "healthcare service" [All Fields] OR "healthcare services" [All Fields] OR "health care service" [All Fields] OR "health care services" [All Fields] OR "quality assurance, health care" [MeSH Terms] OR "quality assurance, health care" [All Fields] OR "performance indicator" [All Fields] OR "performance measure" [All Fields] OR "benchmarking"[MeSH Terms] OR "benchmarking"[All Fields] OR "patient safety" [MeSH Terms] OR "patient safety" [All Fields]
	**OR**
**BLOCK 4** Delivery of Healthcare Block:	"delivery of health care" [MeSH Terms] OR "delivery of health care" [All Fields] OR "delivery of healthcare" [All Fields]
	**OR**
**BLOCK 5** Impact Block:	"affect" [MeSH Terms] OR "affect" [All Fields] OR "effect" [All Fields] OR "Impact" [All Fields] OR "value" [All Fields] OR "influence" [All Fields] OR "change" [All Fields] OR "achieve" [All Fields] OR "induce" [All Fields] OR "improve" [All Fields] OR "progress" [All Fields] OR "increase" [All Fields] OR "help" [All Fields] OR "positive" [All Fields] OR "develop" [All Fields] OR "better" [All Fields] OR "advance" [All Fields] OR "raise" [All Fields] OR "rise" [All Fields] OR "enhance" [All Fields] OR "reinforce" [All Fields] OR "decrease" [All Fields] OR "negative" [All Fields] OR "reduce" [All Fields] OR "reduction" [All Fields] OR "evaluate" [All Fields] OR "evaluation" [All Fields] OR "assess" [All Fields] OR "association" [MeSH Terms] OR "association" [All Fields] OR "relationship" [All Fields] OR "perception" [MeSH Terms] OR "perception" [All Fields] OR “outcome” [All Fields] OR “result” [All Fields] OR "satisfaction" [All Fields] OR “mortality” [MeSH Terms] OR “mortality” [All Fields] OR “morbidity” [MeSH Terms] OR “morbidity” [All Fields] OR "infections" [MeSH Terms] OR "infections" [All Fields] OR "infection" [All Fields] OR "cost" [All Fields] OR "amount" [All Fields]

PubMed Search		
**Number**	**Query**	**Items found**
#97	(#96 AND "2000/01/01"[PDAT] : "2020/02/18"[PDAT])	10921
#96	(#95 AND "loattrfull text"[sb])	12612
#95	(#93 AND #94)	20533
#94	(#12 AND #24)	21095
#93	(#25 OR #26 OR #27 OR #28 OR #29 OR #30 OR #31 OR #32 OR #33 OR #34 OR #35 OR #36 OR #37 OR #38 OR #39 OR #40 OR #41 OR #42 OR #43 OR #44 OR #45 OR #46 OR #47 OR #48 OR #49 OR #50 OR #51 OR #52 OR #53 OR #54 OR #55 OR #56 OR #57 OR #58 OR #59 OR #60 OR #61 OR #62 OR #63 OR #64 OR #65 OR #66 OR #67 OR #68 OR #69 OR #70 OR #71 OR #72 OR #73 OR #74 OR #75 OR #76 OR #77 OR #78 OR #79 OR #80 OR #81 OR #82 OR #83 OR #84 OR #85 OR #86 OR #87 OR #88 OR #89 OR #90 OR #91 OR #92)	19570769
#92	"amount"	456376
#91	"cost"	507909
#90	"infection"	1185251
#89	"infections"	1227804
#88	"infections" [MeSH Terms]	2553387
#87	"morbidity"	373844
#86	"morbidity" [MeSH Terms]	540961
#85	"mortality"	1112437
#84	"mortality" [MeSH Terms]	373200
#83	"satisfaction"	198590
#82	"result"	1031568
#81	"outcome"	1853562
#80	"perception"	354316
#79	"perception" [MeSH Terms]	422892
#78	"relationship"	1570896
#77	"association"	1181565
#76	"association" [MeSH Terms]	13930
#75	"assess"	976277
#74	"evaluation"	1651379
#73	"evaluate"	1147959
#72	"reduction"	1169478
#71	"reduce"	631918
#70	"negative"	1062041
#69	"decrease"	921369
#68	"reinforce"	20315
#67	"enhance"	322306
#66	"rise"	241971
#65	"raise"	62281
#64	"advance"	80772

#63	"better"	939723
#62	"develop"	547934
#61	"positive"	1584415
#60	"help"	507877
#59	"increase"	2000500
#58	"progress"	244645
#57	"improve"	826509
#56	"induce"	425393
#55	"achieve"	275368
#54	"change"	1078363
#53	"influence"	1003326
#52	"value"	1103320
#51	"Impact"	931314
#50	"effect"	3219094
#49	"affect"	679752
#48	"affect" [MeSH Terms]	32649
#47	"delivery of healthcare"	685
#46	"delivery of health care"	106756
#45	"delivery of health care" [MeSH Terms]	1053699
#44	"patient safety"	45153
#43	"patient safety" [MeSH Terms]	19027
#42	"benchmarking"	18539
#41	"benchmarking" [MeSH Terms]	13064
#40	"performance measure"	1811
#39	"performance indicator"	669
#38	"quality assurance, health care"	55567
#37	"quality assurance, health care" [MeSH Terms]	322401
#36	"health care services"	14496
#35	"health care service"	2184
#34	"healthcare services"	8367
#33	"healthcare service"	1694
#32	"health service"	72327
#31	"health services"	449705
#30	"health services" [MeSH Terms]	2090027
#29	"quality improvement"	51990
#28	"quality improvement" [MeSH Terms]	23988
#27	"quality"	1186046
#26	"quality of health care"	74500
#25	"quality of health care" [MeSH Terms]	6783666
#24	(#13 OR #14 OR #15 OR #16 OR #17 OR #18 OR #19 OR #20 OR #21 OR #22 OR #23)	8676996
#23	"healthcare organizations"	11047
#22	"health organizations"	2277

#21	"facilities"	148741
#20	"facility"	130035
#19	"centres"	69312
#18	"centers"	337488
#17	"centre"	1332088
#16	"center"	3556422
#15	"hospital"	4637033
#14	"hospitals"	543591
#13	"hospitals" [MeSH Terms]	269793
#12	(#1 OR #2 OR #3 OR #4 OR #5 OR #6 OR #7 OR #8 OR #9 OR #10 OR #11)	34242
#11	"JCAHO"	1380
#10	"Joint Commission on Accreditation of Hospitals"	133
#9	"joint commission on accreditation of healthcare organizations"	7823
#8	"Joint Commission on Accreditation of Healthcare Organizations" [MeSH Terms]	7449
#7	"accreditations"	77
#6	"accredited"	7907
#5	"accredit"	237
#4	"accredits"	91
#3	"accrediting"	923
#2	"accreditation"	28232
#1	"accreditation" [MeSH Terms]	18685

**Key**	
[MeSH Terms]	Indexing term [medical subject heading (MeSH).
" "	Phrase search.
loattrfull text[sb]	Full text publications [subset]
AND, OR	Boolean operators
[PDAT]	Publication date

2) Cumulative Index to Nursing and Allied Health Literature (CINAHL)

- Searched via EBSCOhost (http://www.ebscohost.com/)
- Searched on 18 February 2020.
- Records retrieved: 2050 … *all results (2050) were from academic journals.*

#	Query	Results
S43	S42 Limiters – Academic Journals	2,050
S42	S41 Limiters – Research Article	2,195
S41	S38 AND S39 Limiters - Published Date: 2000/01-2020/02	9,927
S40	S38 AND S39	10,961
S39	S5 AND S20	14,685
S38	S21 OR S22 OR S23 OR S24 OR S25 OR S26 OR S27 OR S28 OR S29 OR S30 OR S31 OR S32 OR S33 OR S34 OR S35 OR S36 OR S37	3,406,473
S37	TI "assess*" OR AB "assess*" OR TI "association" OR AB "association" OR TI "relation*" OR AB "relation*" OR TI "satisfaction" OR AB "satisfaction" OR TI "cost" OR AB "cost" OR TI "result*" OR AB "result*"	2,011,637
S36	(MH "Outcomes (Health Care)")	47,601
S35	TI "decrease" OR AB "decrease" OR TI "negative" OR AB "negative" OR TI "reduce" OR AB "reduce" OR TI "reduction" OR AB "reduction"	508,069
S34	TI "induce" OR AB "induce" OR TI "improve" OR AB "improve" OR TI "progress" OR AB "progress" OR TI "increase" OR AB "increase" OR TI "help" OR AB "help" OR TI "positive" OR AB "positive" OR TI "develop" OR AB "develop" OR TI "better" OR AB "better" OR TI "advance" OR AB "advance" OR TI "raise" OR AB "raise" OR TI "rise" OR AB "rise" OR TI "enhance" OR AB "enhance" OR TI "reinforce" OR AB "reinforce"	1,155,649
S33	TI "Impact" OR AB "Impact" OR TI "value" OR AB "value" OR TI "influence" OR AB "influence" OR TI "change" OR AB "change" OR TI "achieve" OR AB "achieve"	753,784
S32	(MH "affect") OR (MH "evaluation") OR (MH "perception") OR (MH "mortality") OR (MH "morbidity") OR (MH "infection")	88,903
S31	TX health* N2 delivery	71,091
S30	(MH "health care delivery")	46,741
S29	TX health* N2 service*	560,360
S28	TX "performance indicator*" OR "performance measure*" OR benchmark*	20,564
S27	TI "quality" OR AB "quality"	320,743
S26	(MH "health services")	12,225
S25	(MH "quality assessment")	7,346
S24	(MH "quality assurance")	19,499

S23	(MH "patient safety+")	110,458
S22	(MH "quality improvement+")	58,537
S21	(MH "quality of health care+")	709,370
S20	S6 OR S7 OR S8 OR S9 OR S10 OR S11 OR S12 OR S13 OR S14 OR S15 OR S16 OR S17 OR S18 OR S19	2,211,610
S19	TI ((university or academic) N2 medical N2 (city OR center OR centers OR centre OR centres OR institute* OR facilit*)) OR AB ((university or academic) N2 medical N2 (city OR center OR centers OR centre OR centres OR institute* OR facilit*))	10,206
S18	TI ((cancer or oncology) N2 (center OR centers OR centre OR centres OR institute* OR facilit*)) OR AB ((cancer or oncology) N2 (center OR centers OR centre OR centres OR institute* OR facilit*))	14,596
S17	TI ((cardiac or heart or cardio*) N2 (center OR centers OR centre OR centres OR institute* OR facilit*)) OR AB ((cardiac or heart or cardio*) N2 (center OR centers OR centre OR centres OR institute* OR facilit*))	2,662
S16	TI (stroke N2 (center OR centers OR centre OR centres OR institute* OR facilit*)) OR AB (stroke N2 (center OR centers OR centre OR centres OR institute* OR facilit*))	3,811
S15	TI (rehabilitation N2 (center OR centers OR centre OR centres OR institute* OR facilit*)) OR AB (rehabilitation N2 (center OR centers OR centre OR centres OR institute* OR facilit*))	5,929
S14	TI ((mental or psychiatry*) N2 (center OR centers OR centre OR centres OR institute* OR facilit*)) OR AB ((mental or psychiatry*) N2 (center OR centers OR centre OR centres OR institute* OR facilit*))	3,066
S13	TI (("long term" or geriatric) N2 (center OR centers OR centre OR centres OR institute* OR facilit*)) OR AB (("long term" or geriatric) N2 (center OR centers OR centre OR centres OR institute* OR facilit*))	5,783
S12	TI (p#ediatric N2 (center OR centers OR centre OR centres OR institute* OR facilit*)) OR AB (p#ediatric N2 (center OR centers OR centre OR centres OR institute* OR facilit*))	3,297
S11	TI (trauma N2 (center OR centers OR centre OR centres OR institute* OR facilit*)) OR AB (trauma N2 (center OR centers OR centre OR centres OR institute* OR facilit*))	8,146
S10	TI (birth* N2 (center or centers or centre or centres or institute* or facilit*)) OR AB (birth* N2 (center or centers or centre or centres or institute* or facilit*))	1,514
S9	TX ("health* facilit*") OR "facilities" OR ("health* organization*") OR "facility"	164,707
S8	TX "center" OR "centers" OR "centre" OR "centres"	1,106,616
S7	TX "hospital" OR "hospitals"	1,385,400
S6	(MH "Hospitals+")	105,846
S5	S1 OR S2 OR S3 OR S4	30,605

S4	TX ("Joint Commission on Accreditation of Healthcare Organizations") OR ("Joint Commission on Accreditation of Hospitals") OR "JCAHO"	4,865
S3	(MH "Joint Commission")	8,515
S2	TX "accredit*"	24,265
S1	(MH "accreditation+")	17,397

**Key**	
MH	indexing term (CINAHL heading).
+	subject heading exploded
*	truncation
#	optional wildcard (stands for 0 or 1 character).
TI	terms in the title
AB	terms in the abstract
" "	phrase search
N2	terms within two words of each other (any order).
TX	all text – search of all the database's searchable fields
AND, OR	Boolean operators

3) PsycINFO

- Searched via EBSCOhost (www.ebscohost.com/).
- Date range searched: January 2000 to February 2020.
- Searched on 18 February 2020.
- Records retrieved: 1729 … *all results (1729) were from academic journals.*

#	Query	Results
S45	S8 AND S14 AND S42<br>**Limiters** - Publication Year: 2000-2020; Published Date: 2000/01/01-2020/02/31;<br>**Limiters** - Publication Type: All Journals, Peer Reviewed Journal, Peer-Reviewed Status-Unknown; Exclude Dissertations<br>**Expanders** - Apply equivalent subjects	1,729
S44	S8 AND S14 AND S42	2,362
S43	S8 AND S14	2,598
S42	S15 OR S16 OR S17 OR S18 OR S19 OR S20 OR S21 OR S22 OR S23 OR S24 OR S25 OR S26 OR S27 OR S28 OR S29 OR S30 OR S31 OR S32 OR S33 OR S34 OR S35 OR S36 OR S37 OR S38 OR S39 OR S40 OR S41	3,546,324
S41	TI "assess*" OR AB "assess*" OR TI "association" OR AB "association" OR TI "relation*" OR AB "relation*" OR TI "satisfaction" OR AB "satisfaction" OR TI "cost" OR AB "cost" OR TI "result*" OR AB "result*"	2,622,202
S40	TX "outcome"	234,392
S39	DE "Satisfaction" OR DE "Client Satisfaction" OR DE "Consumer Satisfaction" OR DE "Job Satisfaction" OR DE "Life Satisfaction" OR DE "Need Satisfaction" OR DE "Role Satisfaction"	53,738
S38	DE "Achievement" OR DE "Perception"	50,154
S37	TI "Impact" OR AB "Impact" OR TI "value*" OR AB "value*" OR TI "influence" OR AB "influence" OR TI "achieve*" OR AB "achieve*" OR TI "affect" OR AB "affect" OR TI "infection" OR AB "infection"	1,151,719
S36	TI "decrease" OR AB "decrease" OR TI "negative" OR AB "negative" OR TI "reduce" OR AB "reduce" OR TI "reduction" OR AB "reduction" OR TI "help" OR AB "help" OR TI "positive" OR AB "positive" OR TI "develop" OR AB "develop" OR TI "better" OR AB "better" OR TI "advance" OR AB "advance" OR TI "raise" OR AB	1,306,326

	"raise" OR TI "rise" OR AB "rise" OR TI "enhance" OR AB "enhance" OR TI "reinforce" OR AB "reinforce"	
S35	TI "induce" OR AB "induce" OR TI "improve" OR AB "improve" OR TI "progress" OR AB "progress" OR TI "increase" OR AB "increase"	492,128
S34	SU "morbidity"	8,278
S33	TX "performance indicator*" OR "performance measure*" OR "benchmark*"	17,613
S32	SU "Organi*ational Change"	10,012
S31	DE "Mortality Rate" OR DE "Mortality Risk"	7,369
S30	DE "Evaluation" OR DE "Clinical Audits" OR DE "Program Evaluation" OR DE "Risk Assessment"	66,688
S29	SU Evaluation	144,598
S28	DE "Health Care Services" OR DE "Continuum of Care" OR DE "Health Care Delivery" OR DE "Hospital Programs" OR DE "Long Term Care" OR DE "Mental Health Services" OR DE "Palliative Care"	115,768
S27	DE "Health Care Delivery" OR DE "Health Care Access" OR DE "Health Care Costs" OR DE "Health Care Reform" OR DE "Health Care Utilization" OR DE "Managed Care"	49,462
S26	SU "Health Care Delivery"	20,717
S25	TI quality OR AB quality	248,014
S24	SU "health care services"	44,471
S23	TX "quality assurance"	5,264
S22	SU "Patient Safety"	2,839
S21	SU "Quality control"	2,195
S20	SU "Quality of service"	213
S19	TX "quality improvement"	5,884
S18	SU "Quality of Care"	13,472
S17	TX "quality of health care" or "quality of care" or "healthcare quality"	25,977
S16	TX quality of health care	19,515
S15	DE "Quality Control" OR DE "Quality of Care" OR DE "Quality of Services"	20,766

S14	S9 OR S10 OR S11 OR S12 OR S13	1,067,918
S13	TX "health* facilit*" OR "facilities" OR "health* organization*" OR "facility"	65,146
S12	TI ((mental or psychiatry*) N2 (center OR centers OR centre OR centres OR institute* OR facilit*)) OR AB ((mental or psychiatry*) N2 (center OR centers OR centre OR centres OR institute* OR facilit*))	11,390
S11	TX center* OR centre*	737,582
S10	TX "hospital*"	449,902
S9	DE "Hospitals" OR DE "Psychiatric Hospitals"	23,326
S8	S1 OR S2 OR S3 OR S4 OR S5 OR S6 OR S7	8,304
S7	TX JCAHO	76
S6	TX "Joint Commission on Accreditation of Healthcare Organi*ations"	137
S5	SU "hospital accreditation"	107
S4	TX "Joint Commission"	562
S3	TX accredit*	7,987
S2	TX accreditation	5,241
S1	SU accreditation	1,938

**Key**	
DE	descriptors (subjects [exact]), specific subject terms
SU	subject headings
*	truncation
TI	terms in the title
AB	terms in the abstract
" "	phrase search
N2	terms within two words of each other (any order).
TX	all text – search of all the database's searchable fields
AND, OR	Boolean operators

4) EMBASE 1974 to 2020 Week 07

- Searched via Ovid (http://ovidsp.ovid.com/).
- Date range searched: 2000 to current.
- Searched on 18 February 2020.
- Records retrieved: 3316

Number	Searches	Results
1	exp accreditation/	60645
2	accreditation$1.mp.	42604
3	accredit$.tw.	30170
4	(joint commission or joint commission on accreditation of hospital$1 or joint commission on accreditation of healthcare organi#ations or jcaho or jcia).mp.	5747
5	or/1-4	78917
6	exp hospital/	1094146
7	hospital$1.mp.	2210382
8	(center$1 or centre$1).mp.	1339390
9	healthcare organi#ation$.tw.	6793
10	health$ institution$.mp.	6853
11	exp health care facility/	1480729
12	health$ facilit$.tw.	23106
13	((university or academic) adj medical adj (city or center$1 or centre$1 or health facilit$)).tw.	35347
14	((cancer or oncology) adj2 (center$1 or centre$1 or institut$ or facilit$)).tw.	79556
15	((cardiac or heart or cardio$) adj2 (center$1 or centre$1 or institut$ or facilit$)).tw.	18603
16	(stroke adj2 (center$1 or centre$1 or institut$ or facilit$)).tw.	6394
17	(rehabilitation adj2 (center$1 or centre$1 or institut$ or facilit$)).tw.	15818
18	((mental or psychiatry$2) adj2 (center$1 or centre$1 or institut$ or facilit$)).tw.	10502
19	((long term or geriatric) adj2 (center$1 or centre$1 or institut$ or facilit$)).tw.	13740
20	(p?ediatric adj2 (center$1 or centre$1 or institut$ or facilit$)).tw.	14622
21	(trauma adj2 (center$1 or centre$1 or institut$ or facilit$)).tw.	21895

22	(birth$ adj2 (center$1 or centre$1 or institut$ or facilit$)).tw.	2298
23	or/6-22	3687282
24	exp health care quality/	3108457
25	(quality of health care or health care quality).mp.	242265
26	exp total quality management/	62115
27	total quality management.mp.	62717
28	quality improve$.mp.	58561
29	quality control/ or quality of life/	629064
30	health$ service$1.mp.	557756
31	quality of service$.mp.	7811
32	quality assurance, health care.mp.	189
33	quality assurance.tw.	35962
34	quality indicator$.tw.	11748
35	quality measure$.tw.	10479
36	performance indicator$1.tw.	5178
37	bench?mark$.tw.	47106
38	patient safety.mp. or exp patient safety/	135557
39	exp health care delivery/	3139292
40	(delivery of health care or health care delivery).mp.	178113
41	exp satisfaction/	230680
42	satisfaction.mp. or job satisfaction/ or patient satisfaction/	276759
43	exp mortality/	1038794
44	mortality risk/ or mortality.mp. or mortality rate/	1419649
45	morbidity.mp. or exp morbidity/	644247
46	infection/ or infection rate/ or infection.mp.	2259910
47	(impact or affect or value or outcome$1 or perception$1).mp.	6481956
48	exp health personnel attitude/	179453
49	(influence or effect or change or result or cost or evaluat$ or "assess" or association or relation$).tw.	14722150
50	(decrease or negative or reduce or reduction).tw.	4338861
51	(achieve$ or induce$1 or improve$ or progress or increase or help or positive or develop$ or better or advance$ or raise or rise or enhance$ or reinforce).tw.	14981588
52	or/24-51	24060984
53	5 and 23	29868

54	52 and 53	29595
55	**limit** 54 to yr="2000 -current"	23564
56	**limit** 55 to full text	3316

**Key**	
/	indexing term (Emtree heading).
exp	exploded indexing term (Emtree heading).
$	Truncation.
ti,ab	terms in either title or abstract fields.
tw	text word search in title or abstract fields.
?	optional wildcard (stands for 0 or 1 character).
#	mandated wildcard (stands for exactly 1 character).
adj2	terms within two words of each other (any order).
mp	multi-purpose – searches in title, original title, abstract, subject heading, name of substance and registry word fields.
" "	phrase search.
AND, OR	Boolean operators

5) MEDLINE

- Searched via Ovid (http://ovidsp.ovid.com/).
- Database: Ovid MEDLINE(R) 1946 to February 14, 2020
- Date range searched: 2000 to current.
- Searched on 18 February 2020.
- Records retrieved: 1407

Number	Searches	Results
1	exp Accreditation/	18693
2	accreditation$1.mp.	25389
3	accredit$.tw.	18455
4	exp Joint Commission on Accreditation of Healthcare Organizations/	7451
5	(joint commission or joint commission on accreditation of hospital$1 or joint commission on accreditation of healthcare organi#ations or jcaho or jcia).mp.	9157
6	or/1-5	31103
7	exp hospitals/	269911
8	hospital$1.mp.	1239780
9	(center$1 or centre$1).mp.	727729
10	health$ institution$.mp.	4330
11	health$ facilit$.tw.	14634
12	healthcare organi#ation$.tw.	5268
13	((university or academic) adj medical adj (city or center$1 or centre$1 or health facilit$)).tw.	19731
14	((cancer or oncology) adj2 (center$1 or centre$1 or institut$ or facilit$)).tw.	36658
15	((cardiac or heart or cardio$) adj2 (center$1 or centre$1 or institut$ or facilit$)).tw.	8333
16	(stroke adj2 (center$1 or centre$1 or institut$ or facilit$)).tw.	1971
17	(rehabilitation adj2 (center$1 or centre$1 or institut$ or facilit$)).tw.	8765
18	((mental or psychiatry$2) adj2 (center$1 or centre$1 or institut$ or facilit$)).tw.	6711
19	(p?ediatric adj2 (center$1 or centre$1 or institut$ or facilit$)).tw.	6944
20	(trauma adj2 (center$1 or centre$1 or institut$ or facilit$)).tw.	14350
21	(birth$ adj2 (center$1 or centre$1 or institut$ or facilit$)).tw.	1659
22	((long term or geriatric) adj2 (center$1 or centre$1 or institut$ or facilit$)).tw.	9157

23	or/7-22	1869979
24	exp "Quality of Health Care"/	6785035
25	(quality of health care or health care quality).mp.	141179
26	exp Quality Improvement/	24052
27	quality improve$.mp.	44442
28	exp Quality Control/	48603
29	quality control.mp.	74209
30	exp Total Quality Management/	12490
31	total quality management.mp.	12882
32	exp Quality Assurance, Health Care/	322605
33	(quality assurance, health care or quality assurance).mp.	68576
34	exp Health Services/	2091081
35	health$ service$1.mp.	417663
36	quality of service$.mp.	4766
37	quality indicator$.tw.	6430
38	performance indicator$1.tw.	2878
39	bench?mark$.tw.	26729
40	exp Patient Safety/	19060
41	patient safety.mp.	36414
42	exp Delivery of Health Care/	1054687
43	job satisfaction.mp. or exp Job Satisfaction/	26302
44	satisfaction.mp. or exp Personal Satisfaction/	177327
45	patient satisfaction.mp. or exp Patient Satisfaction/	99017
46	exp Mortality/	373564
47	mortality risk/ or mortality.mp. or mortality rate/	1014378
48	morbidity.mp. or exp Morbidity/	812895
49	infection/ or infection rate/ or infection.mp.	1047368
50	Attitude of Health Personnel.mp. or exp "Attitude of Health Personnel"/	155621
51	(impact or affect or value or outcome$1 or perception$1).mp.	4226102
52	(influence or effect or change or result or cost or evaluat$ or "assess" or association or relation$).tw.	8747992
53	(decrease or negative or reduce or reduction).tw.	2786204

54	(achieve$ or induce$1 or improve$ or progress or increase or help or positive or develop$ or better or advance$ or raise or rise or enhance$ or reinforce).tw.	10065815
55	or/24-54	17740273
56	6 and 23	12218
57	55 and 56	12096
58	limit 57 to yr="2000 -current"	7513
59	limit 58 to full text	1407

**Key**	
/	indexing term (MeSH heading).
exp	exploded indexing term (MeSH heading).
$	truncation.
ti,ab	terms in either title or abstract fields.
tw	text word search in title or abstract fields.
?	optional wildcard (stands for 0 or 1 character).
#	mandated wildcard (stands for exactly 1 character).
adj2	terms within two words of each other (any order).
mp	multi-purpose – searches in title, original title, abstract, subject heading, name of substance and registry word fields.
" "	phrase search.
AND, OR	Boolean operators

6) Cochrane Database of Systematic Reviews (CDSR)
7) The Cochrane Central Register of Controlled Trials (CENTRAL)

- Searched via Cochrane Library (https://cochranelibrary.com/)
- Issue 2 of 12, February 2020
- Searched on 18 February 2020
- Records retrieved: 567 (71 CDSR, 496 CENTRAL).

ID	Searches	Hits
#1	MeSH descriptor: [Accreditation] explode all trees	23
#2	MeSH descriptor: [Joint Commission on Accreditation of Healthcare Organizations] explode all trees	3
#3	accredit*	1079
#4	"joint commission" or "joint commission on accreditation of hospital*" or "joint commission on accreditation of healthcare organi?ations" or "jcaho" or "jcia"	173
#5	#1 OR #2 OR #3 OR #4	1213
#6	MeSH descriptor: [Hospitals] explode all trees	3541
#7	MeSH descriptor: [Health Facilities] explode all trees	14093
#8	MeSH descriptor: [Rehabilitation Centers] this term only	308
#9	MeSH descriptor: [Academic Medical Centers] this term only	340
#10	MeSH descriptor: [Birthing Centers] this term only	14
#11	MeSH descriptor: [Maternal-Child Health Centers] this term only	46
#12	MeSH descriptor: [Trauma Centers] this term only	183
#13	MeSH descriptor: [Secondary Care Centers] this term only	7
#14	MeSH descriptor: [Tertiary Care Centers] this term only	301
#15	Hospital*:ti,ab,kw	171745
#16	(center or centers or centre or centres):ti,ab,kw	122555
#17	(Health* NEAR/2 institution*):ti,ab,kw	404
#18	((university or academic) NEAR/2 medical NEAR/2 (city or center or centers or centre or centres or "health facilit*")):ti,ab,kw	3740
#19	((cancer or oncology or cardiac or heart or cardio* or stroke or rehabilitation or mental or psychiatry* or "long term" or geriatric or p*ediatric or trauma or birth*) NEAR/2 (center or centers or centre or centres or institut* or facilit*)):ti,ab,kw	13976
#20	#6 OR #7 OR #8 OR #9 OR #10 OR #11 OR #12 OR #13 OR #14 OR #15 OR #16 OR #17 OR #18 OR #19	273799
#21	#5 AND #20	585

#23	#5 AND #20 (with Cochrane Library publication date from Jan 2000 to Feb 2020, in **Cochrane Reviews and Cochrane Protocols**)	71
#24	#5 AND #20 (with Publication Year from 2000 to 2020, **in Trials**)	496

**Key**	
MeSH descriptor	indexing term [medical subject heading (MeSH)].
*	truncation
ti,ab,kw	terms in either title or abstract or keyword fields.
NEAR/2	terms within two words of each other (any order).
" "	phrase search.
AND, OR	Boolean operators

8) Social Sciences Citation Index (SSCI)

- Searched via Web of Science (www.webofknowledge.com/).
- Date range searched: 2000 to 2020.
- Searched on 18 February 2020.
- Records retrieved: 1588

Set	Results	Search
# 34	1,588	(#31 AND #30) AND **DOCUMENT TYPES**: (Article OR Abstract of Published Item OR Database Review OR Review)<br>*Indexes=SSCI Timespan=2000-2020*
# 33	1,650	#31 AND #30<br>*Indexes=SSCI Timespan=2000-2020*
# 32	1,791	#31 AND #30<br>*Indexes=SSCI Timespan=All years*
# 31	1,864	#20 AND #4<br>*Indexes=SSCI Timespan=All years*
# 30	3,813,911	#29 OR #28 OR #27 OR #26 OR #25 OR #24 OR #23 OR #22 OR #21<br>*Indexes=SSCI Timespan=All years*
# 29	3,799,534	TS=(mortality OR morbidity OR infection* OR impact OR affect OR value OR outcome* OR perception* OR influence OR effect OR change OR result OR cost OR evaluat* OR association OR relation* OR decrease OR negative OR reduce OR reduction OR achieve* OR induce* OR improve* OR progress OR increase OR help OR positive OR develop* OR better OR advance* OR raise OR rise OR enhance* OR reinforce OR attitude)<br>*Indexes=SSCI Timespan=All years*
# 28	128,711	TS=(satisfaction OR "job satisfaction" OR "patient satisfaction")<br>*Indexes=SSCI Timespan=All years*
# 27	4,965	TS=("delivery of health care" OR "health care delivery")<br>*Indexes=SSCI Timespan=All years*
# 26	10,775	TS=("patient safety")<br>*Indexes=SSCI Timespan=All years*
# 25	39,459	TS=("quality indicator*" OR "performance indicator*" OR "quality measure*" OR "performance measure*" OR benchmarking OR "bench-mark" OR "bench-marking" OR "bench marking")<br>*Indexes=SSCI Timespan=All years*
# 24	2,354	TS=("quality of service*")<br>*Indexes=SSCI Timespan=All years*
# 23	68,570	TS=("health* service*")<br>*Indexes=SSCI Timespan=All years*
# 22	9,245	TS=("total quality management" OR "quality control" OR "quality assurance")<br>*Indexes=SSCI Timespan=All years*
# 21	3,801	TS=("quality of health care" OR "health care quality")<br>*Indexes=SSCI Timespan=All years*
# 20	341,287	#19 OR #18 OR #17 OR #16 OR #15 OR #14 OR #13 OR #12 OR #11 OR #10 OR #9 OR #8 OR #7 OR #6 OR #5<br>*Indexes=SSCI Timespan=All years*

# 19	968	TS=(birth* NEAR/2 (center OR centers OR centre OR centres OR institute* OR facilit*)) *Indexes=SSCI Timespan=All years*
# 18	2,141	TS=(trauma NEAR/2 (center OR centers OR centre OR centres OR institute* OR facilit*)) *Indexes=SSCI Timespan=All years*
# 17	1,007	TS=((pediatric OR paediatric) NEAR/2 (center OR centers OR centre OR centres OR institute* OR facilit*)) *Indexes=SSCI Timespan=All years*
# 16	3,909	TS=(("long term" OR geriatric) NEAR/2 (center OR centers OR centre OR centres OR institute* OR facilit*)) *Indexes=SSCI Timespan=All years*
# 15	5,044	TS=((mental OR psychiatry*) NEAR/2 (center OR centers OR centre OR centres OR institute* OR facilit*)) *Indexes=SSCI Timespan=All years*
# 14	3,615	TS=(rehabilitation NEAR/2 (center OR centers OR centre OR centres OR institute* OR facilit*)) *Indexes=SSCI Timespan=All years*
# 13	543	TS=(stroke NEAR/2 (center OR centers OR centre OR centres OR institute* OR facilit*)) *Indexes=SSCI Timespan=All years*
# 12	629	TS=((cardiac OR heart OR cardio*) NEAR/2 (center OR centers OR centre OR centres OR institute* OR facilit*)) *Indexes=SSCI Timespan=All years*
# 11	5,197	TS=((cancer OR oncology) NEAR/2 (center OR centers OR centre OR centres or institute* OR facilit*)) *Indexes=SSCI Timespan=All years*
# 10	4,658	TS=((university OR academic) NEAR/2 medical NEAR/2 (city OR center* OR centre* OR "health facility*")) *Indexes=SSCI Timespan=All years*
# 9	2,373	TS=("health* facility") *Indexes=SSCI Timespan=All years*
# 8	1,876	TS=("health* institut*") *Indexes=SSCI Timespan=All years*
# 7	14,465	TS=("health* organization*") *Indexes=SSCI Timespan=All years*
# 6	182,131	TS=(center OR centers OR centre OR centres) *Indexes=SSCI Timespan=All years*
# 5	156,921	TS=(hospital OR hospitals) *Indexes=SSCI Timespan=All years*
# 4	7,245	#3 OR #2 OR #1 *Indexes=SSCI Timespan=All years*
# 3	650	TS=("joint commission" OR "joint commission on accreditation of hospital*" OR "joint commission on accreditation of healthcare organizations" OR "jcaho" OR "jcia") *Indexes=SSCI Timespan=All years*
# 2	6,842	TS=(accredit*) *Indexes=SSCI Timespan=All years*
# 1	4,664	TS=(accreditation) *Indexes=SSCI Timespan=All years*

9) KCI-Korean Journal Database

- Searched via Web of Science (www.webofknowledge.com/).
- Date range searched: 2000 to 2020.
- Searched on 18 February 2020.
- Records retrieved: 421

Set	Results	Search
# 34	421	(#33) AND **DOCUMENT TYPES:** (Research-Article)<br>*Indexes=KJD Timespan=2000-2020*
# 33	421	#32<br>*Indexes=KJD Timespan=2000-2020*
# 32	421	#31 AND #30<br>*Indexes=KJD Timespan=All years*
# 31	433	#20 AND #4<br>*Indexes=KJD Timespan=All years*
# 30	1,283,270	#29 OR #28 OR #27 OR #26 OR #25 OR #24 OR #23 OR #22 OR #21<br>*Indexes=KJD Timespan=All years*
# 29	1,281,738	TS=(mortality OR morbidity OR infection* OR impact OR affect OR value OR outcome* OR perception* OR influence OR effect OR change OR result OR cost OR evaluat* OR association OR relation* OR decrease OR negative OR reduce OR reduction OR achieve* OR induce* OR improve* OR progress OR increase OR help OR positive OR develop* OR better OR advance* OR raise OR rise OR enhance* OR reinforce OR attitude)<br>*Indexes=KJD Timespan=All years*
# 28	54,319	TS=(satisfaction OR "job satisfaction" OR "patient satisfaction")<br>*Indexes=KJD Timespan=All years*
# 27	133	TS=("delivery of health care" OR "health care delivery")<br>*Indexes=KJD Timespan=All years*
# 26	584	TS=("patient safety")<br>*Indexes=KJD Timespan=All years*
# 25	6,861	TS=("quality indicator*" OR "performance indicator*" OR "quality measure*" OR "performance measure*" OR benchmarking OR "bench-mark" OR "bench-marking" OR "bench marking")<br>*Indexes=KJD Timespan=All years*
# 24	1,913	TS=("quality of service*")<br>*Indexes=KJD Timespan=All years*
# 23	1,890	TS=("health* service*")<br>*Indexes=KJD Timespan=All years*
# 22	3,141	TS=("total quality management" OR "quality control" OR "quality assurance")<br>*Indexes=KJD Timespan=All years*
# 21	148	TS=("quality of health care" OR "health care quality")<br>*Indexes=KJD Timespan=All years*
# 20	106,994	#19 OR #18 OR #17 OR #16 OR #15 OR #14 OR #13 OR #12 OR #11 OR #10 OR #9 OR #8 OR #7 OR #6 OR #5<br>*Indexes=KJD Timespan=All years*

# 19	49	TS=(birth* NEAR/2 (center OR centers OR centre OR centres OR institute* OR facilit*)) *Indexes=KJD Timespan=All years*
# 18	138	TS=(trauma NEAR/2 (center OR centers OR centre OR centres OR institute* OR facilit*)) *Indexes=KJD Timespan=All years*
# 17	37	TS=((pediatric OR paediatric) NEAR/2 (center OR centers OR centre OR centres OR institute* OR facilit*)) *Indexes=KJD Timespan=All years*
# 16	628	TS=(("long term" OR geriatric) NEAR/2 (center OR centers OR centre OR centres OR institute* OR facilit*)) *Indexes=KJD Timespan=All years*
# 15	438	TS=((mental OR psychiatry*) NEAR/2 (center OR centers OR centre OR centres OR institute* OR facilit*)) *Indexes=KJD Timespan=All years*
# 14	744	TS=(rehabilitation NEAR/2 (center OR centers OR centre OR centres OR institute* OR facilit*)) *Indexes=KJD Timespan=All years*
# 13	283	TS=(stroke NEAR/2 (center OR centers OR centre OR centres OR institute* OR facilit*)) *Indexes=KJD Timespan=All years*
# 12	154	TS=((cardiac OR heart OR cardio*) NEAR/2 (center OR centers OR centre OR centres OR institute* OR facilit*)) *Indexes=KJD Timespan=All years*
# 11	572	TS=((cancer OR oncology) NEAR/2 (center OR centers OR centre OR centres OR institute* OR facilit*)) *Indexes=KJD Timespan=All years*
# 10	579	TS=((university OR academic) NEAR/2 medical NEAR/2 (city OR center* OR centre* OR "health facility*")) *Indexes=KJD Timespan=All years*
# 9	103	TS=("health* facility") *Indexes=KJD Timespan=All years*
# 8	155	TS=("health* institut*") *Indexes=KJD Timespan=All years*
# 7	1,051	TS=("health* organization*") *Indexes=KJD Timespan=All years*
# 6	78,265	TS=(center OR centers OR centre OR centres) *Indexes=KJD Timespan=All years*
# 5	30,323	TS=(hospital OR hospitals) *Indexes=KJD Timespan=All years*
# 4	1,564	#3 OR #2 OR #1 *Indexes=KJD Timespan=All years*
# 3	92	TS=("joint commission" OR "joint commission on accreditation of hospital*" OR "joint commission on accreditation of healthcare organizations" OR "jcaho" OR "jcia") *Indexes=KJD Timespan=All years*
# 2	1,483	TS=(accredit*) *Indexes=KJD Timespan=All years*
# 1	1,107	TS=(accreditation) *Indexes=KJD Timespan=All years*

10) Russian Science Citation Index (RSCI)

- Searched via Web of Science (www.webofknowledge.com/).
- Date range searched: 2005 to 2020 (all years).
- Searched on 18 February 2020.
- Records retrieved: 38

Note: all years in RSCI are (2005-2020)

Set	Results	Search
# 22	38	(#21) AND **DOCUMENT TYPES:** (Article OR Review)<br>*Indexes=RSCI Timespan=All years*
# 21	41	#20 AND #4<br>*Indexes=RSCI Timespan=All years*
# 20	25,420	#19 OR #18 OR #17 OR #16 OR #15 OR #14 OR #13 OR #12 OR #11 OR #10 OR #9 OR #8 OR #7 OR #6 OR #5<br>*Indexes=RSCI Timespan=All years*
# 19	6	TS=(birth* NEAR/2 (center OR centers OR centre OR centres OR institute* OR facilit*))<br>*Indexes=RSCI Timespan=All years*
# 18	17	TS=(trauma NEAR/2 (center OR centers OR centre OR centres OR institute* OR facilit*))<br>*Indexes=RSCI Timespan=All years*
# 17	107	TS=((pediatric OR paediatric) NEAR/2 (center OR centers OR centre OR centres OR institute* OR facilit*))<br>*Indexes=RSCI Timespan=All years*
# 16	42	TS=(("long term" OR geriatric) NEAR/2 (center OR centers OR centre OR centres OR institute* OR facilit*))<br>*Indexes=RSCI Timespan=All years*
# 15	86	TS=((mental OR psychiatry*) NEAR/2 (center OR centers OR centre OR centres OR institute* OR facilit*))<br>*Indexes=RSCI Timespan=All years*
# 14	179	TS=(rehabilitation NEAR/2 (center OR centers OR centre OR centres OR institute* OR facilit*))<br>*Indexes=RSCI Timespan=All years*
# 13	44	TS=(stroke NEAR/2 (center OR centers OR centre OR centres OR institute* OR facilit*))<br>*Indexes=RSCI Timespan=All years*
# 12	423	TS=((cardiac OR heart OR cardio*) NEAR/2 (center OR centers OR centre OR centres OR institute* OR facilit*))<br>*Indexes=RSCI Timespan=All years*
# 11	386	TS=((cancer or oncology) NEAR/2 (center OR centers OR centre OR centres OR institute* OR facilit*))<br>*Indexes=RSCI Timespan=All years*
# 10	15	TS=((university OR academic) NEAR/2 medical NEAR/2 (city OR center* OR centre* OR "health facility*"))<br>*Indexes=RSCI Timespan=All years*

# 9	44	TS=("health* facility")<br>*Indexes=RSCI Timespan=All years*
# 8	143	TS=("health* institut*")<br>*Indexes=RSCI Timespan=All years*
# 7	416	TS=("health* organization*")<br>*Indexes=RSCI Timespan=All years*
# 6	18,466	TS=(center OR centers OR centre OR centres)<br>*Indexes=RSCI Timespan=All years*
# 5	7,045	TS=(hospital OR hospitals)<br>*Indexes=RSCI Timespan=All years*
# 4	260	#3 OR #2 OR #1<br>*Indexes=RSCI Timespan=All years*
# 3	4	TS=("joint commission" OR "joint commission on accreditation of hospital*" OR "joint commission on accreditation of healthcare organizations" OR "jcaho" OR "jcia")<br>*Indexes=RSCI Timespan=All years*
# 2	256	TS=(accredit*)<br>*Indexes=RSCI Timespan=All years*
# 1	185	TS=(accreditation)<br>*Indexes=RSCI Timespan=All years*

11) SciELO Citation Index

- Searched via Web of Science (www.webofknowledge.com/).
- Date range searched: 2002 to 2020 (all years).
- Searched on 18 February 2020.
- Records retrieved: 250

Note: all years in SciELO Citation Index are (2002-2020)

Set	Results	Search
# 22	250	(#21) AND **DOCUMENT TYPES:** (Research-Article OR Review-Article OR Case-Report OR Undefined)<br>*Indexes=SCIELO Timespan=All years*
# 21	262	#20 AND #4<br>*Indexes=SCIELO Timespan=All years*
# 20	66,655	#19 OR #18 OR #17 OR #16 OR #15 OR #14 OR #13 OR #12 OR #11 OR #10 OR #9 OR #8 OR #7 OR #6 OR #5<br>*Indexes=SCIELO Timespan=All years*
# 19	77	TS=(birth* NEAR/2 (center OR centers OR centre OR centres OR institute* OR facilit*))<br>*Indexes=SCIELO Timespan=All years*
# 18	128	TS=(trauma NEAR/2 (center OR centers OR centre OR centres OR institute* OR facilit*))<br>*Indexes=SCIELO Timespan=All years*
# 17	166	TS=((pediatric OR paediatric) NEAR/2 (center OR centers OR centre OR centres OR institute* OR facilit*))<br>*Indexes=SCIELO Timespan=All years*
# 16	134	TS=(("long term" OR geriatric) NEAR/2 (center OR centers OR centre OR centres OR institute* OR facilit*))<br>*Indexes=SCIELO Timespan=All years*
# 15	285	TS=((mental OR psychiatry*) NEAR/2 (center OR centers OR centre OR centres OR institute* OR facilit*))<br>*Indexes=SCIELO Timespan=All years*
# 14	286	TS=(rehabilitation NEAR/2 (center OR centers OR centre OR centres OR institute* OR facilit*))<br>*Indexes=SCIELO Timespan=All years*
# 13	69	TS=(stroke NEAR/2 (center OR centers OR centre OR centres OR institute* OR facilit*))<br>*Indexes=SCIELO Timespan=All years*
# 12	330	TS=((cardiac OR heart OR cardio*) NEAR/2 (center OR centers OR centre OR centres OR institute* OR facilit*))<br>*Indexes=SCIELO Timespan=All years*
# 11	632	TS=((cancer OR oncology) NEAR/2 (center OR centers OR centre OR centres OR institute* OR facilit*))<br>*Indexes=SCIELO Timespan=All years*
# 10	66	TS=((university OR academic) NEAR/2 medical NEAR/2 (city OR center* OR centre* OR "health facility*"))<br>*Indexes=SCIELO Timespan=All years*

# 9	244	TS=("health* facility")<br>*Indexes=SCIELO Timespan=All years*
# 8	1,151	TS=("health* institut*")<br>*Indexes=SCIELO Timespan=All years*
# 7	3,464	TS=("health* organization*")<br>*Indexes=SCIELO Timespan=All years*
# 6	27,786	TS=(center OR centers OR centre OR centres)<br>*Indexes=SCIELO Timespan=All years*
# 5	38,242	TS=(hospital OR hospitals)<br>*Indexes=SCIELO Timespan=All years*
# 4	898	#3 OR #2 OR #1<br>*Indexes=SCIELO Timespan=All years*
# 3	24	TS=("joint commission" OR "joint commission on accreditation of hospital*" OR "joint commission on accreditation of healthcare organizations" OR "jcaho" OR "jcia")<br>*Indexes=SCIELO Timespan=All years*
# 2	884	TS=(accredit*)<br>*Indexes=SCIELO Timespan=All years*
# 1	621	TS=(accreditation)<br>*Indexes=SCIELO Timespan=All years*

**Web of Science Key**	
TS	topic tag; searches terms in title, abstract, author keywords and keywords plus fields.
*	truncation
NEAR/2	terms within two words of each other (any order).
" "	phrase search.
AND, OR	Boolean operators

12) ScienceDirect

- Searched via ScienceDirect (http://sciencedirect.com/).
- Date range searched: 2000 to 2020.
- Searched on 18 February 2020.
- Records retrieved: 1772

- Searching was conducted for title, abstract, or key words through research articles, review articles, data articles, and case reports (2000-2020)

Accredit, hospital – 751 results
Accredit, centers – 729 results
Joint Commission, hospital – 236 results
Joint Commission, center – 299 results
Accreditation, impact – 528 results
Accreditation, outcome – 749 results
Accreditation, quality – 1391 results
**Total exported references 4683**
**After removing duplicates 1772**

13) PROSPERO

- Searched via www.crd.york.ac.uk/PROSPERO/.
- Searched on 18 February 2020.
- Records retrieved: 169.

Number	Searches	Results
#1	MeSH DESCRIPTOR accreditation EXPLODE ALL TREES	3
#2	accreditation	68
#3	accredited	89
#4	accredit*:ti,kw	5
#5	MeSH DESCRIPTOR Joint Commission on Accreditation of Healthcare Organizations EXPLODE ALL TREES	0
#6	Joint Commission on Accreditation of Healthcare Organizations	2
#7	joint commission	15
#8	JCAHO or jcia	3
#9	external assessment or external evaluation	12
#10	#1 OR #2 OR #3 OR #4 OR #5 OR #6 OR #7 OR #8 OR #9	169

**Key**	
MeSH descriptor	indexing term [medical subject heading (MeSH)].
*	truncation
ti,kw	terms in either title or keyword fields.
AND, OR	Boolean operators
