# Supplementary material for: The impact of hospital accreditation on the quality of healthcare: a systematic literature review
Source: BMC Health Serv Res. 2021 Oct 6;21:1057. doi: 10.1186/s12913-021-07097-6 (PMC8493726; doi:10.1186/s12913-021-07097-6)
Supplement: Supplementary file 3 — Additional file 3: Summary of the key findings of all studies included in the review (n = 76). [file 12913_2021_7097_MOESM3_ESM.pdf]

Additional File 3:
Summary of the key findings of all studies included in the review (n=76).

Author, Country	Study Objective	Study Design	Main Results	Number of Hospitals (accreditation body - Country)	Impact (Category)*	Overall Methodological Quality
**Lám[56] 2016, Hungary** *(in Hungarian)*	Assess organizational culture changes in pilot institutes of the accreditation program.	Descriptive, comparative, cross-sectional research using before-and-after design.	The increase in the proportion of positive responses was statistically significant in the dimensions of organizational learning, continuous improvement, open communication, and teamwork in the organizational unit, whereas none of the decreased dimensions was significant.	4 (BELLA - Hungary)	Positive (1)	**Fair**
**Lee[57] 2016, Korea**	Compare registered nurses' perceptions of safety climate and attitude toward medication error reporting before and after hospital accreditation and identify the relationship between perceived safety climate and attitude toward medication error reporting.	A comparative descriptive longitudinal questionnaire-based study using before-and-after design.	The level of safety climate increased significantly after accreditation. Out of 19 items in the survey, 12 items were improved significantly. Similarly, the attitude toward medication error reporting increased, and negative attitudes decreased significantly after accreditation.	1 (KOIHA - Korea)	Positive (1)	**Fair**
**Andres[58] 2019, China**	Assess the longitudinal relationship between accreditation and hospital professional staff perception of organizational culture.	A prospective exploratory longitudinal cohort study design.	The hierarchical culture was the dominant organizational culture domain pre-accreditation. Following accreditation, hierarchical culture declined significantly but remained dominant, while group and developmental culture increased.	1 (ACHS - Australia)	Positive (1)	**Fair**
**Kim[59] 2019, Korea** *(in Korean)*	Compare nurse accreditation perception, job stress, turnover intention, safety management recognition in a general hospital before and after hospital's accreditation.	A descriptive comparative study with a before-and-after design.	There was no significant difference in turnover intention and safety management culture before and after accreditation. Compared to the pre-accreditation preparatory phase, job stress decreased significantly after accreditation.	1 (KOIHA - Korea)	Neutral (1) Negative (2)	**Fair**
**Greenfield[60] 2019, Australia**	Examine the effect of longitudinal accreditation participation on improving continuity of quality patient care and human resource management (HRM) processes outcomes.	Cohort longitudinal study through secondary data analysis. Data were collected in 2003-2006 (Time 1) and 2007-2010 (Time 2).	The results for HRM processes score at Time 1 and Time 2 were: T1 (2.92 ± 0.29) and T2 (3.10 ± 0.26). HRM processes scores and the continuity of quality patient care significantly improved over time for all three of the mandatory accreditation performance groups.	311 (ACHS - Australia)	Positive (1, 5)	**Good**
**Domingues[49] 2017, Brazil**	Evaluate the influence of hospital accreditation on the process of permanent education in Health.	A cross-sectional, descriptive, retrospective, documentary, and quantitative research carried out using a before-and-after study design.	Overall, there was evidence of a gradual increase in the number of educational activities by 22% post-accreditation compared to pre-accreditation (56 vs 44 activities, respectively). The multi-professional team had an increase of 20% in the number of educational activities, and the nursing team had an increase of 37% as recipients.	1 (ONA - Brazil)	Positive (2)	**Poor**

Author, Country	Study Objective	Study Design	Main Results	Number of Hospitals (accreditation body - Country)	Impact (Category)*	Overall Methodological Quality
**Al-Faouri[61] 2019, Jordan**	Assess healthcare providers' perceived stress levels before and after hospital accreditation survey site visits.	A cross-sectional descriptive study using a before-and-after design.	Perceived stress levels among healthcare providers were significantly higher before accreditation site visits compared to post-accreditation.	2 (HCAC - Jordan)	Negative (2)	**Good**
**Higashi[62] 2013, Brazil**	Evaluate the frequency of perceived work-related stressors by nurses in hospitals based on accreditation status.	A cross-sectional questionnaire-based study.	At an accredited hospital, nurses perceived more work-related stressors compared to non-accredited ones.	3 (ONA - Brazil)	Negative (2)	**Fair**
**Elkins[63] 2010, USA**	Examine hospital accreditation impact on the perceived stress among nursing hospital management and administrative employees.	A descriptive observational cross-sectional questionnaire-based study using a before-and-after research design.	Perceived stress, anxiety, and depression were significantly higher during accreditation preparation compared to post-accreditation. Staff satisfaction and sleep function were improved significantly post-accreditation compared to the accreditation preparation period.	1 (JCAHO - USA)	Negative (2)	**Fair**
**Kagan[64] 2016, Israel**	Examine the long-term impact of hospital accreditation on the nursing work environment and the association between nursing work environment and the perceived accreditation on organizational climate.	A longitudinal before-and-after questionnaire-based study.	Post-accreditation scores in both samples; the nonpaired (n=763) and paired (n=89), for the 3 components of the nursing work environment (i.e., autonomy, control over nursing practice, and nurse-physician relations) were non-significantly higher than the pre-accreditation scores, except for autonomy subscale in the paired sample.	1 (JCIA - USA)	Neutral (2)	**Fair**
**Oliveira[65] 2018, Brazil**	Analyze the influence of hospital accreditation on the work environment of the nursing team.	Cross-sectional study using an explanatory sequential mixed-method approach.	There was no statistically significant association between hospital accreditation and nursing staff perception of the work environment, either at pairs level (private accredited vs private non-accredited, private accredited vs public non-accredited) and in evaluating the three groups together.	3 (ONA - Brazil)	Neutral (2)	**Fair**
**Oliveira[66] 2016, Brazil**	Compare and identify the nursing working environment of accredited and non-accredited public hospitals.	Quantitative cross-sectional study.	Hospital accreditation resulted in no significant effect on nurses' work environment as the environment was favorable to all NWI-R domains.	2 (ONA - Brazil)	Neutral (2)	**Good**
**Oliveira[67] 2019, Brazil**	Analyze the influence of hospital accreditation on the professional satisfaction of nursing workers.	Multicentric cross-sectional study using an explanatory mixed-method approach.	Overall, workers of the accredited hospital had a better job satisfaction score. The comparison of the three groups investigated confirmed that accreditation positively influenced the professional satisfaction of the nursing workers.	3 (ONA - Brazil)	Positive (2)	**Good**
**Um[68] 2018, Korea**	Examine clinical nutrition service provisions based on hospital accreditation status.	A comparative nationwide cross-sectional survey.	Malnourished patients in accredited hospitals received an insignificantly higher nutritional intervention rate compared to non-accredited hospitals. In accredited hospitals, time spent on direct care was significantly higher, however, it was significantly lower for outpatient care compared to non-accredited hospitals.	35 (JCIA - USA)	Neutral (2, 5)	**Fair**

Author, Country	Study Objective	Study Design	Main Results	Number of Hospitals (accreditation body - Country)	Impact (Category)*	Overall Methodological Quality
**Joseph[55] 2018, India**	Examine the impact of hospital accreditation on physical infrastructure and patient satisfaction in public hospitals.	Quantitative cross-sectional survey-based study.	There was no significant impact of accreditation on patient satisfaction (mean score 4.28 in both accredited and non-accredited hospitals).	14 (NABH and KASH - India)	Neutral (3)	**Poor**
**Heuer[69] 2004, USA**	Examine the relationship between accreditation scores and independently measured patient satisfaction scores.	A quasi-experimental study using Ex post facto comparative retrospective analysis design.	There was no relationship between summative accreditation scores and patient satisfaction and no relationship between patient satisfaction and corresponding Joint Commission assessment categories.	41 (JCAHO - USA)	Neutral (3)	**Fair**
**Hayati[70] 2010, Malaysia**	Examine the impact of hospital accreditation on patient satisfaction and assess the association between patient satisfaction and hospital workload.	A cross-sectional comparative study.	There was no significant difference in patients' satisfaction with the provided quality of services between accredited and non-accredited hospitals neither at overall level nor at dimensions level.	4 (MSQH - Malaysia)	Neutral (3)	**Fair**
**Barghouthi[71] 2018, Palestine**	Assess the relationship between patient satisfaction and hospital accreditation status.	A quantitative descriptive cross-sectional comparative questionnaire-based study.	The mean of patient satisfaction was slightly higher in non-accredited hospitals compared to accredited hospitals; however, the difference was not significant.	2 (JCIA - USA)	Neutral (3)	**Fair**
**Haj-Ali[72] 2014, Lebanon**	Explore the impact of hospital accreditation on patient satisfaction.	An observational explanatory cross-sectional questionnaire-based study.	Most of the patients (76.34%) were unsatisfied with the quality of services. After confounders adjustment, there was no statistically significant association between accreditation classification and patient satisfaction.	6 (MoPH - Lebanon)	Neutral (3)	**Good**
**Al-Qahtani[73] 2012, Saudi Arabia**	Examine hospital accreditation impact on the quality of health care services in Obstetrics and Gynecology clinics, as perceived by patients.	A cross-sectional comparative questionnaire-based study.	Although patients at accredited and non-accredited hospitals had positive perceptions of the provided quality of services, patients at the accredited hospital were more satisfied.	2 (JCIA - USA)	Positive (3)	**Good**
**Ajarmah[74] 2015, Jordan**	Examine the effect of hospital accreditation programs on patient satisfaction.	Descriptive quantitative cross-sectional study, questionnaire-based.	There was a significant difference in patients' satisfaction with the provided quality of services between accredited and non-accredited hospitals at the overall level and all SERVQUAL dimensions level.	74 (not reported)	Positive (3)	**Fair**
**Mohebbifar[75] 2017, Iran**	Examine the relationship between hospital accreditation and patient satisfaction.	A cross-sectional descriptive questionnaire-based study.	Overall, patients had a moderate level of satisfaction toward the quality of health care services provided (65%). After adjusting for confounders, hospital accreditation had a significant inverse association with overall patient satisfaction.	7 (MOHME - Iran)	Negative (3)	**Fair**

Author, Country	Study Objective	Study Design	Main Results	Number of Hospitals (accreditation body - Country)	Impact (Category)*	Overall Methodological Quality
**Andres[76] 2019, China**	Assess the longitudinal impact of hospital accreditation on patient experience in a university teaching hospital.	A prospective exploratory longitudinal study design. Patient experience was assessed by three cross-sectional surveys.	Cross-sectionally, all domains of patient experience scores declined (improved) over the study period. Regression analysis confirmed declining (improving) all parameter estimates 3 months and 15 months following accreditation except for the 'continuity and transition' domain.	1 (ACHS - Australia)	Positive (3)	**Good**
**Sack[77] 2010, Germany**	Assess the relationship between patient satisfaction and accreditation status in the field of cardiology.	Comparative descriptive cross-sectional study.	There was no statistically significant difference between accredited and non-accredited units regarding the "recommendation rate of a given hospital"	25 (KTQ and pCC - Germany)	Neutral (3)	**Good**
**Sack[78] 2011, Germany**	Assess the relationship between patient satisfaction and accreditation status.	Comparative descriptive cross-sectional study.	Overall, 66.3% of all the patients recommend their hospital to others. This recommendation, however, was not related to the accreditation status in the univariate analyses.	73 (KTQ and pCC - Germany)	Neutral (3)	**Good**
**Lam[21] 2018, USA**	Comparing health outcomes of patients admitted to US accredited hospitals versus hospitals reviewed by state surveys, and whether Joint Commission accreditation associated with an additional benefit for patients compared with other independent accrediting bodies.	Observational cross-sectional retrospective study.	Patients treated at accredited hospitals had lower 30-day mortality rates than those at hospitals that were reviewed by a state survey agency, but nearly identical mortality rates for surgical conditions. Medical conditions readmissions at 30 days were significantly lower at accredited hospitals than at state survey hospitals but did not differ for the surgical conditions. Patient experience scores were modestly better at state survey hospitals than at accredited hospitals.	4400 (JCAHO and other independent bodies - USA)	Negative (3) Positive (4)	**Good**
**Marzban[53] 2017, Iran**	Examine the statuses of quality indices in 11 accredited educational hospitals with different accreditation degrees.	Descriptive cross-sectional study.	There was no significant difference in performance indicators between hospitals with different accreditation degrees.	11 (MOHME - Iran)	Neutral (3, 4)	**Poor**
**Salmon[48] 2003, South Africa**	Assess the effects of COHSASA accreditation program on public hospitals' processes and outcomes in a developing country setting.	Prospective randomized control trial.	Intervention hospitals improved their average overall compliance with COHSASA standards significantly from 48% to 78% in about 2 years after accreditation began. No meaningful change occurred in any service element in the control hospitals. Apart from the nurses' perceptions of clinical quality in the accredited hospitals, the remaining seven quality indicators showed little or no effect; patient satisfaction with care; patient medication education; accessibility of medical records; completeness of medical records; completeness of perioperative notes; labeling of ward stock; and, composite assessment of hospital sanitation.	20 (COHSASA - South Africa)	Neutral (3, 5)	**Fair**

Author, Country	Study Objective	Study Design	Main Results	Number of Hospitals (accreditation body - Country)	Impact (Category)*	Overall Methodological Quality
**Salim[25] 2017, United Arab Emirates**	Assess the impact of accreditation on Infection Control (IC) performance measures.	An empirical longitudinal case study.	Overall, accreditation showed a potentially positive impact on infection control measures. The trend of VAP, CLABSI, and CAUTI was declining in the period before accreditation. The VAP and CLABSI significantly dropped immediately post-accreditation, whereas CAUTI level significantly increased. The level of SSI also decreased non-significantly after the JCI accreditation in 2010 while the trend was increasing before.	1 (JCIA - USA)	Positive (4)	**Fair**
**Mørk Hansen[50] 2013, Denmark** *(in Danish)*	Evaluate whether the accreditation process has resulted in a decrease in the prevalence of nosocomial infections in the Region of Northern Jutland, Denmark.	A retrospective prevalence study.	Pre-accreditation, the overall prevalence of nosocomial infections was slightly insignificantly increasing, while in the period parallel to the accreditation process the overall prevalence decreased significantly. There were no statistically significant changes in the prevalence of PNEU, DSSI, and SE/BA pre-accreditation, but an annual increase in the prevalence of UTIs of 16.6% was statistically significant.	5 (DDKM - Denmark)	Positive (4)	**Poor**
**Almasabi[52] 2017, Saudi Arabia**	Understand the impact of CBAHI accreditation on the quality of care in Saudi Arabia.	Mixed methods approach, quantitative and qualitative (i.e., cross-sectional surveys, documentary analyses, and interviews).	Quantitatively, accreditation has no significant effect on mortality in the three hospitals, in addition to conflicting findings in terms of infection rate and length of stay.	3 (CBAHI - Saudi Arabia)	Neutral (4)	**Poor**
**Barnett[80] 2017, USA**	Assess whether heightened vigilance during survey weeks is associated with a change in patient outcomes compared with non-survey weeks.	Observational quasi-randomized analysis.	There was a significant decrease in 30-day mortality for admissions occurring during a survey week vs. the surrounding 3 weeks. Larger effects were observed among major teaching hospitals.	1984 (JCAHO - USA)	Positive (4)	**Good**
**Towers[81] 2014, USA**	Explore the possibility of the ebb and flow in patient outcomes with Joint Commission accreditation site visits by examining monthly patterns in risk-adjusted inpatient mortality rates around accreditation site visits. As well, explore the role of slack resources in shielding healthcare organizations from the ebbs and flows of external pressures.	A retrospective cohort study.	Adherence to Joint Commission standards contributes to improved inpatient mortality rates in hospitals. Mortality rates temporarily dropped significantly following Joint Commission inspection at Month (+1) period, and subsequently, return to pre-inspection levels (an approximately 4% reduction in the risk-adjusted mortality rate).	58 (JCAHO - USA)	Positive (4)	**Fair**
**Falstie-Jensen[82] 2015, Denmark**	Examine the association between hospital accreditation compliance and mortality.	A prospective follow-up population-based study.	Compared to patients at partially accredited hospitals, patients at fully accredited hospitals (i.e., high compliance with accreditation standards) had a lower 30-days mortality risk.	31 (DDKM - Denmark)	Positive (4)	**Good**

Author, Country	Study Objective	Study Design	Main Results	Number of Hospitals (accreditation body - Country)	Impact (Category)*	Overall Methodological Quality
**Falstie-Jensen[84] 2018, Denmark**	Examine the association between compliance with consecutive accreditation cycles and patient outcomes (namely, mortality, length of stay, and acute readmission).	A prospective observational follow-up population-based study.	Patients admitted at persistent low compliant hospitals had a higher 30-day mortality risk, a longer length of stay, whereas acute readmission had no difference than patients at high compliant hospitals.	25 (DDKM - Denmark)	Positive (4)	**Good**
**Miller[85] 2005, USA**	Examine the association between JCAHO accreditation scores and AHRQ Inpatient Quality Indicators (IQIs) and the Patient Safety Indicators (PSIs).	A descriptive-analytical retrospective study.	Despite variation in AHRQ performance measures, most hospitals scored high (90% and 100%) on JCAHO measures with no significant relationship between them. None of the IQIs appeared to be related to the final overall evaluation score (FOES) at the $P < 0.05$.	2116 (JCAHO - USA)	Neutral (4)	**Good**
**Arab[86] 2017, Iran** *(in Persian)*	Investigate the impact of hospital accreditation process on healthcare outcomes performance indicators.	A quasi-experimental longitudinal descriptive-analytical study using an Interrupted Time Series design.	After hospital accreditation implementation, the average length of stay and bed turnover rate decreased significantly, patient satisfaction and the bed occupancy rate increased significantly, and the cesarean section rate decreased insignificantly compared to the pre-accreditation period.	14 (MOHME - Iran)	Positive (4)	**Fair**
**Wardhani[88] 2019, Indonesia**	Explore the association of hospital design factors, market competition, and performance with hospital accreditation status.	An observational comparative study.	Although the differences in performance between accredited and non-accredited hospitals were not significant, accredited hospitals tended to have a higher bed occupancy rate, the average length of stay, turnover interval, net mortality rate, and gross mortality rate than not-accredited hospitals.	346 (ICAH - Indonesia)	Neutral (4)	**Fair**
**Falstie-Jensen[89] 2015, Denmark**	Examine the association between hospital compliance with accreditation and length of stay (LOS) and acute readmission (AR).	A cohort retrospective nationwide population-based follow-up study.	Admissions at fully accredited hospitals were associated with a modest significantly shorter LOS compared with admissions at partially accredited hospitals after adjusting for confounding factors. Collaterally, no difference was observed in AR within 30 days after discharge.	31 (DDKM - Denmark)	Positive (4)	**Good**
**Janati[91] 2016, Iran**	Assess the impact of hospital accreditation on ICU quality of care and patient safety measures.	An interventional observational study.	Introducing accreditation interventions resulted in reducing pressure ulcer incidence significantly by 2.7% during the study period. similarly, a reduction of 1.45 days in the ICU average length of stay was significant. In contrast, an unexpected increase in hospital-acquired infection from 1.5% to 8.1% was seen.	1 (MOHME - Iran)	Positive (4)	**Fair**

Author, Country	Study Objective	Study Design	Main Results	Number of Hospitals (accreditation body - Country)	Impact (Category)*	Overall Methodological Quality
**Mumford[92] 2015, Australia**	Investigate the use of Staphylococcus aureus bacteremia (SAB) rates as an outcome indicator to measure the benefits of accreditation.	A retrospective cohort study.	Staphylococcus aureus bacteremia (SAB) rates across all hospitals fell from 1.34 per 10 000 bed days in 2009 to 0.77 per 10 000 bed days in 2012. Higher performing hospitals – in terms of higher accreditation scores, and especially in terms of infection control scores – were not associated with lower SAB rates.	77 (ACHS - Australia)	Neutral (4)	**Fair**
**Jarrah[93] 2019, Jordan**	Explore the impact of hospital accreditation on patient safety in terms of defining triggers and adverse events.	Descriptive comparative record-based study.	Overall, hospital accreditation resulted in improving patient care and decrease adverse events. Hospital length of stay was significantly lower in accredited hospitals compared to non-accredited ones. Although the number of triggers in the accredited hospitals was significantly higher compared to non-accredited ones, the number of adverse events was significantly less.	6 (HCAC - Jordan)	Positive (4)	**Fair**
**Leite[94] 2019, Brazil**	Compare Coronary Care Unit (CCU) length of stay (LOS) and the total hospital LOS of Acute Coronary Syndrome (ACS) patients before and after ONA 3 accreditation (excellence). Additionally, examine the impact of accreditation on clinical outcomes.	A systematic, observational, descriptive, prospective study.	CCU LOS reduced non-significantly from 3 to 2,5 days. Regarding the hospital LOS, there was a significant reduction from 8 to 6 days. In ACS subgroups, there was a significant reduction only at the hospital LOS in non-STEMI patients.	1 (ONA - Brazil)	Positive (4)	**Good**
**Al-Sughayir[95] 2016, Saudi Arabia**	Investigate whether hospital accreditation drives improvements for the length of stay in psychiatric inpatients.	Non-experimental observational study through retrospective medical records review.	The average length of stay (LOS) post-accreditation was significantly reduced compared to the pre-accreditation period.	1 (ACI - Canada)	Positive (4)	**Fair**
**Petrović[8] 2018, Serbia**	Investigate the impact of hospital accreditation process on health care quality indicators values.	Quasi-experimental comparative study of the "Difference-in-Difference" (DiD).	The accreditation process had a significant positive effect attributed to a shorter length of waiting for the first scheduled health cheque and surgical cheque, lower decubitus rate, and a decrease in length of stay for acute myocardial infarction patients, whereas mortality rate, mortality within the first 48 hours, and the average length of stay were not affected.	2 (AZUS - Serbia)	Positive (4, 5)	**Fair**
**Chen[79] 2003, USA**	Examine the association between hospital accreditation, quality of care (i.e., use of recommended guidelines), and survival among acute myocardial infarction hospitalized patients.	A cross-sectional comparative study.	Patients treated at JCAHO surveyed hospitals had significantly lower 30-day mortality, were more likely to receive aspirin and beta-blockers, both on admission and during hospitalization, and reperfusion therapy, than patients treated at non-surveyed hospitals.	4221 (JCAHO - USA)	Positive (4, 5)	**Fair**

Author, Country	Study Objective	Study Design	Main Results	Number of Hospitals (accreditation body - Country)	Impact (Category)*	Overall Methodological Quality
**Al-Awa[51] 2011, Saudi Arabia**	This study aimed to determine if the accreditation process has a positive impact on patient safety and quality of care.	The interventional study, 4-year retrospective, and prospective study design.	Compared to pre-accreditation, 27 (33%) performance indicators out of 81 showed significant improvement post-accreditation. These indicators were in the following areas: mortality, Healthcare-Associated Infections (HAI), cardiopulmonary resuscitation codes, surgeries and invasive procedures, blood transfusion reaction, and adverse events.	1 (ACI - Canada)	Positive (4, 5)	**Poor**
**Mosadeghrad[87] 2018, Iran** *(in Persian)*	Examine the association between hospital accreditation and hospital performance measures. In addition to identifying and ranking accreditation constructs that most affecting hospital performance.	A descriptive correlational study using data on two cross-sections.	Hospitals' accreditation scores were not correlated with their performance measures. However, accreditation scores of leadership and management; accident and emergency; and procurement departments had the most effect on hospitals' performance while physiotherapy, environmental health, and nutrition were the least.	244 (MOHME - Iran)	Neutral (4, 5)	**Fair**
**Halasa[90] 2015, Jordan**	Assess the economic impact of hospital accreditation on selected structural and outcome performance measures.	A retrospective observational comparative study using difference-in-differences (DiD).	Compared to baseline data, accreditation resulted in a 119.3% improvement in the quality index. Explicitly, significant improvements were seen in the reduction in patients who returned to the ICU, reduction in annual staff turnover, and improvement in the completeness of medical records. These improvements translated into total savings of US$ 593 000 over 3 years. Hospital readmission within 30 days and return to surgery within 24 hours were not significant.	4 (JCIA - USA)	Positive (4, 5, 6)	**Good**
**Griffith[83] 2002, USA**	Examine the relationship of seven outcomes performance measures in non-federal general hospitals against Joint Commission scores.	Descriptive comparative correlational study.	Generally, Joint Commission measures were not correlated with outcome measures. The few significant correlations that appear are often counterintuitive.	742 (JCAHO - USA)	Neutral (4, 6)	**Fair**
**Shaw[19] 2010, Belgium, Czech, France, Ireland, UK, Poland, Spain**	Identify systematic differences in quality management systems between hospitals that were accredited, or certificated, or neither.	A descriptive comparative study.	Overall compliance scores for 229 quality and safety criteria were 66.9% in accredited, 60.0% in certified, and 51.2% in hospitals neither accredited nor certified. Compliance score with quality and safety measures was consistently higher in accredited hospitals, except for the 'patients' rights' dimension.	71 (not reported)	Positive (5)	**Fair**
**Shaw[28] 2014, Czech, France, Germany, Poland, Spain, Portugal, Turkey**	Explore the relationship between ISO 9001 certification, healthcare accreditation, and quality management activities in European hospitals.	Cross-sectional, mixed-method, and multi-level study.	In the clinical practice domain, accreditation showed benefits in AMI and stroke more than in deliveries and hip fracture; the most significant impact was the clinical review in stroke. Accreditation promotes processes and structural elements but has a limited effect on evidence-based practice delivery.	73 (not reported)	Positive (5)	**Good**

Author, Country	Study Objective	Study Design	Main Results	Number of Hospitals (accreditation body - Country)	Impact (Category)*	Overall Methodological Quality
**Abedi[54] 2014, Iran**	Examine the impact of hospital accreditation on reported medical errors.	A descriptive comparative study using a before-and-after design.	There was no significant difference in the rate of reported medical errors after accreditation compared to before accreditation.	38 (not reported)	Neutral (5)	**Poor**
**Devkaran[96] 2015, United Arab Emirates**	Examine the impact of healthcare accreditation on hospital quality measures.	Interrupted time series (ITS), longitudinal quasi-experimental design.	Preparation for accreditation survey resulted in improvement as 20 of the 27 measures had a positive pre-accreditation slope (thirteen out of which are statistically significant). Additionally, accreditation had no significant impact (either positive or negative) on 11 out of the 27 measures. However, a residual benefit from accreditation, in terms of sustaining performance, was achieved during accreditation preparation (i.e., 20% higher than the baseline level in 2009).	1 (JCIA - USA)	Positive (5)	**Good**
**Falstie-Jensen[97] 2017, Denmark**	Examine the association between accreditation compliance and delivering recommended hospital care.	A prospective follow-up population-based study.	Patients at fully accredited hospitals (i.e., high compliance with accreditation standards) were more likely to receive the recommended care for stroke, COPD, diabetes, and hip fracture than patients treated at partially accredited hospitals. In contrast, heart failure patients had an inverse association, whereas ulcer patients had no difference.	31 (DDKM - Denmark)	Positive (5)	**Good**
**Devkaran[98] 2019, United Arab Emirates**	Evaluate hospital re-accreditation impact on quality, patient safety, and reliability over three accreditation cycles by testing the accreditation life cycle model on quality measures.	Interrupted time series (ITS), longitudinal quasi-experimental design.	Levene's test demonstrated a significant reduction in variation of the composite score quality measures with subsequent accreditation cycles. The study showed that repeated surveys could reduce variations and sustain improvements over the accreditation cycle.	1 (JCIA - USA)	Positive (5)	**Good**
**Bogh[99] 2016, Denmark**	Evaluate the impact of the first accreditation cycle in Denmark on changes over time in the quality of hospital care in all Danish public hospitals.	A multi-level, longitudinal, stepped-wedge, prospective, nationwide study.	When all performance measures were included in the analysis, a positive non-significant change was observed during accreditation compared with the period before accreditation. However, restricting the analyses to care processes that did not meet the target (compliance 68%) before accreditation, revealed a significant positive change in trend.	25 (DDKM - Denmark)	Positive (5)	**Good**
**Schmaltz[100] 2011, USA**	Examine the association between Joint Commission accreditation and hospital performance measures for common diseases.	Observational comparative analysis study.	Compared to non-accredited hospitals, accredited hospitals had better baseline performance, larger gains over time, and significantly higher performance in 2008 on 13 out of 16 performance measures. After adjustment for baseline characteristics, the absolute difference in improvement was 4.2%.	3679 (JCAHO - USA)	Positive (5)	**Good**

Author, Country	Study Objective	Study Design	Main Results	Number of Hospitals (accreditation body - Country)	Impact (Category)*	Overall Methodological Quality
**Mumford[101] 2014, Australia**	Investigate hand hygiene suitability as an indicator of accreditation outcomes and test the positive correlation hypothesis between better accreditation outcomes and higher hand hygiene compliance score.	A retrospective, longitudinal, multisite comparative survey.	After matching the hand hygiene data with hospitals that underwent two accreditation surveys, achieving full accreditation for both surveys (n=8) was not significantly associated with higher hand hygiene rates versus those hospitals achieving full accreditation in only one survey.	96 (ACHS - Australia)	Neutral (5)	**Fair**
**Barker[102] 2002, USA**	Identify the prevalence of medication administration errors.	A prospective cohort study.	In accredited and non-accredited hospitals, (234/1481) 16% and (56/284) 20% of the doses were in error, respectively. There was no significant difference in error rates by accreditation status.	24 (JCAHO - USA)	Neutral (5)	**Fair**
**Braga[103] 2018, Brazil**	Examine the association between hospital accreditation level and results of care indicators, in addition to analyzing the proposed interventions and anticipate indicators' results for the next five years.	Retrospective quantitative documentary study.	Hospitals with the highest certification level did not have better care indicators results; however, it showed consistency in the results, which suggested a more secure organizational culture.	5 (ONA - Brazil)	Neutral (5)	**Fair**
**Bogh[104] 2015, Denmark**	Examine longitudinal improvement in performance measures based on participation in accreditation programs (accredited vs. non-accredited hospitals).	A historical, prospective follow-up population-based study.	Participating in accreditation was not associated with larger improvement in performance measures as both groups (i.e., accredited and non-accredited) significantly improved their care performance processes over time.	33 (JCIA - USA; HQS - UK)	Neutral (5)	**Good**
**Braithwaite[18] 2010, Australia**	Determine whether accreditation performance is associated with a self-reported clinical performance and independent ratings of four aspects of organizational performance (organizational culture, organizational climate, consumer involvement, and leadership)	Independent blinded assessment of organizational culture, organizational climate, consumer involvement, and leadership.	Accreditation performance was significantly positively correlated with organizational culture and leadership behaviors. Organizational climate and consumer involvement were not significantly associated with accreditation ratings.	19 (ACHS - Australia)	Positive (5)	**Good**
**Lutfiyya[105] 2009, USA**	Determine whether quality measures used in the center of Medicaid and medicare service (CMS) Hospital Compare database differed for rural critical access hospitals based on accreditation status.	A cross-sectional comparative study.	Compared to non-accredited, accredited rural critical access hospitals had significantly better compliance for 4 out of 16 hospital quality indicators (namely; % of AMI patients given aspirin at arrival, % of heart failure patients given ACE inhibitor for LVSD, % of heart failure patients given adult smoking cessation counseling, and % of pneumonia patients - with a history of smoking - given smoking cessation counseling).	730 (JCAHO - USA)	Positive (5)	**Good**
**Al-Sughayir[106] 2014, Saudi Arabia**	Examine if hospital accreditation drives improvements in the clinical practice of giving pro re nata (PRN) antipsychotic medications for psychiatric inpatients.	An observational comparative retrospective record-based study using before-and-after design.	There was a 38% reduction in the number of administered PRN antipsychotics post-accreditation, which was statistically significant.	1 (ACI - Canada)	Positive (5)	**Good**

Author, Country	Study Objective	Study Design	Main Results	Number of Hospitals (accreditation body - Country)	Impact (Category)*	Overall Methodological Quality
**Wang[107] 2015, China**	Discuss the effectiveness of safe medication administration stewardship intervention in inpatient care and provide some reference for international counterparts.	Interventional non-randomized study.	The number of medication administration errors, intravenous administration errors, type 2 errors (errors reached patient and required monitoring and/or intervention to preclude harm), and the number of errors related to high-alert medications decreased post-accreditation significantly compared to pre-accreditation.	1 (JCIA - USA)	Positive (5)	**Good**
**Nomura[108] 2016, Brazil**	Analyze the quality of nursing documentation before and after hospital accreditation preparation, using the Quality of Nursing Diagnoses, Interventions, and Outcomes (Q-DIO) instrument.	An observational comparative retrospective study.	A significant improvement in the quality of nursing documentation was observed in 24 out of the 29 items (82.8%). The improvement was significant in all domains; nursing diagnoses as a process, nursing diagnoses as a product, nursing Interventions, and nursing Outcomes.	1 (JCIA - USA)	Positive (5)	**Fair**
**Habib[109] 2016, Lebanon**	Assess the relationship between accreditation status and compliance with occupational health and safety accreditation standards.	A cross-sectional descriptive study.	Compared to non-accredited hospitals, accredited hospitals reported better occupational health and safety (OHS) performance in almost all indicators. Accredited hospitals were significantly better in training OHS committee members on OHS principles and policies, training hospital staff on office safety, having experienced OHS officers, and having emergency preparedness.	68 (MoPH - Lebanon)	Positive (5)	**Fair**
**Pourreza[110] 2017, Iran** *(in Persian)*	Investigate the impact of compliance with hospital accreditation standards on emergency department performance indicators.	Cross-sectional descriptive-analytical study.	There was no significant relationship between accreditation and the performance of emergency departments in included hospitals.	8 (MOHME - Iran)	Neutral (5)	**Fair**
**Al-Sughayir[111] 2017, Saudi Arabia**	Investigate whether hospital accreditation drives improvements for administered pro re nata (PRN) benzodiazepines in psychiatric inpatients.	Non-experimental observational study through retrospective medical records review.	Accreditation resulted in a reduction of approximately 22% in the number of administered PRN benzodiazepines. The average number of PRN benzodiazepine administrations per patient post-accreditation was significantly reduced compared to the pre-accreditation period.	1 (ACI - Canada)	Positive (5)	**Good**
**Salehian[112] 2015, Iran** *(in Persian)*	Assess the impact of hospital accreditation on productivity indexes in a public teaching hospital.	Interventional analytical cross-sectional study.	Hospital accreditation had a significant positive impact on the income-expense axis, a relatively positive effect on the performance indexes axis and specific unit axis, and no impact on the human resource axis. The amount of insurance deduction, amount on income not paid, and income:cost ratio were improved significantly after accreditation, whereas energy consumption per bed was not affected in the income-expense axis.	1 (MOHME - Iran)	Positive (6)	**Fair**

Author, Country	Study Objective	Study Design	Main Results	Number of Hospitals (accreditation body - Country)	Impact (Category)*	Overall Methodological Quality
**Lindlbauer[113] 2016, Germany**	Examine quality management certification impact on technical efficiency using data envelopment analysis (DEA).	A retrospective descriptive longitudinal study.	The mean efficiency of KTQ accredited hospitals differs significantly from the mean efficiency of non-accredited hospitals. Accreditation had a significant positive net impact on efficiency in the year of accreditation and the following two years and remained positive but insignificant after that.	748 (KTQ - Germany)	Positive (6)	**Good**
**Okumura[114] 2019, Japan**	Examine the impact of standardization of the perioperative protocol for operating time in cataract surgery based on the Joint Commission International (JCI) accreditation guidelines.	A retrospective observational comparative study using a before-and-after design.	Pre-procedure time and post-procedure time significantly decreased after JCI accreditation, while the procedure time did not change substantially. Consequently, the total procedure time significantly reduced on average by 7.3 min per person after JCI accreditation.	1 (JCIA - USA)	Positive (6)	**Good**
**Lin[115] 2019, Taiwan**	Evaluate efficiency utilizing an objective performance assessment of various hospital departments and examine how hospital accreditations contribute toward hospital efficiencies.	Descriptive analytical study.	Impulse Response Function (IRF) revealed that accreditation improved the efficiency of all departments during the first year of accreditation introductions.	15 (TJCHA - Taiwan)	Positive (6)	**Fair**
**Saquetto[116] 2019, Brazil**	Describe the efficiency of private hospitals in Brazil and understand the influence of property structure, specialization, accreditation, and teaching activity on private hospitals' efficiency.	Descriptive analytical cross-section study.	Accreditation has a significant negative impact on hospital efficiency, especially under varying assumptions of scale. Staffing demands and investments in equipment and resources contributed to reducing the efficiency of private hospitals after accreditation.	98 (ONA - Brazil; JCI and NIAHIO - USA; ACI - Canada)	Negative (6)	**Good**
**Inomata[117] 2018, Japan**	Examine JCI accreditation impact on operating room (OR) efficiency by comparing periods for patients who received surgeries (elective and emergency).	A retrospective observational study.	The total procedure/surgery time, procedure/surgery time, and post-procedure time were not significantly different between before-and-after JCI accreditation. The pre-anesthesia time was significantly slightly increased after accreditation, whereas the anesthesia induction time was significantly reduced.	1 (JCIA - USA)	Neutral (6)	**Fair**

* Impact categories: 1) changes in organizational culture and management; 2) changes at professionals' level; 3) changes at the patient level; 4) changes in patient clinical outcomes; 5) changes in the quality of services provided; 6) changes in economic outcomes. ACHS, Australian council on healthcare standards; MOHME, ministry of health and medical education; JCIA, joint commission international accreditation; TJCHA, Taiwan joint commission on hospital accreditation; ACI, accreditation Canada international; NIAHO, national integrated accreditation for healthcare organisations; ONA, national accreditation organisation (in Portuguese: organização nacional dae acreditação); KTQ, cooperation for transparency and quality in hospitals (in German: kooperation für transparenz und qualität im gesundheitswesen); MoPH, ministry of public health; JCAHO, joint commission on accreditation of healthcare organisations; DDKM, Danish healthcare quality programme (in Danish: den danske kvalitetsmodel); HQS, health quality services; AZUS, agency for accreditation of health care institutions of Serbia; HCAC, health care accreditation council; COHSASA, the council for health services accreditation of Southern Africa; KOIHA, Korean institute for healthcare accreditation; CBAHI, Saudi central board for accreditation of healthcare institutions; BELLA, accreditation of healthcare providers for safe patient care (in Hungarian: betegellátók akkreditációja a biztonságos betegellátásért); pCC, procum cert; MSQH, Malaysian society for quality in health; NABH, national accreditation board for hospitals and healthcare providers; KASH, Kerala accreditation standards for hospitals; ICAH, Indonesia commission on accreditation of hospitals
